# Role of DEAD-box RNA helicases in low-temperature adapted growth of Antarctic *Pseudomonas syringae* Lz4W

**DOI:** 10.1128/spectrum.04335-22

**Published:** 2023-11-28

**Authors:** Ashaq Hussain, Malay Kumar Ray

**Affiliations:** 1 Centre for Cellular and Molecular Biology, Hyderabad, Telangana, India; LSU Health New Orleans, New Orleans, Louisiana, USA

**Keywords:** cold adaptation, RNA helicases, gene disruption, homologous recombination, growth analysis, functional complementation

## Abstract

*Pseudomonas syringae* possesses genes for all five major DEAD-box RNA helicases, *rhlE*, *srmB*, *csdA*, *dbpA*, and *rhlB*, that are found in γ-proteobacteria. Bioinformatic analysis of the RNA helicase genes provided detailed insights into their genomic organization, promoter characteristics, regulatory 3′ regions, and sequence similarity. Disruption of *rhlB* and *rhlE* genes did not have any effect on growth of the *P. syringae* mutants at optimum (22°C) or at low (4°C) temperatures. On the other hand, disruption of *srmB* and *dbpA* genes caused a slow-growing phenotype in the mutants at low temperature (4°C). In comparison to the above-mentioned helicases, deletion of *csdA* gene caused the mutant (Δ*csdA*) to be totally incapable of growing at 4°C while marginally affecting the growth (resulting in slow growth) at the optimum temperature (22°C). Functional complementation studies revealed that RNA helicases are functionally non-redundant, as the roles performed by different helicases are individual and specific.

**IMPORTANCE:**

RNA metabolism is important as RNA acts as a link between genomic information and functional biomolecules, thereby playing a critical role in cellular response to environment. We investigated the role of DEAD-box RNA helicases in low-temperature adapted growth of *P. syringae*, as this group of enzymes play an essential role in modulation of RNA secondary structures. This is the first report on the assessment of all major DEAD-box RNA helicases in any Antarctic bacterium. Of the five RNA helicases, three (*srmB*, *csdA*, and *dbpA*) are important for the growth of the Antarctic *P. syringae* at low temperature. However, the requisite role of *dbpA* and the indispensable requirement of *csdA* for low-temperature adapted growth are a novel finding of this study. Growth analysis of combinatorial deletion strains was performed to understand the functional interaction among helicase genes. Similarly, genetic complementation of RNA helicase mutants was conducted for identification of gene redundancy in *P. syringae*.

## INTRODUCTION

RNA helicases regulate cellular processes by modulating different aspects of RNA biology, from its synthesis to degradation, structural maturation (RNA processing) to the stability, and for interaction with metabolites to cellular proteins (RNA protein complexes) for sensing and responding to the cellular milieu (1, 2). The DEAD-box RNA helicases constitute a specific group of RNA helicases that derives its name from the highly conserved amino acid sequence in one of the motifs that reads as DEAD in single-letter amino acid code (3). The DEAD-box proteins belong to the SF2 superfamily of RNA helicases which bind to ATP and RNA by two RecA-like domains located on 350- to 400-residues-long “helicase core” and perform ATP (ATPase)dependent functions (4, 5). These functions include canonical RNA duplex unwinding, RNA strand annealing, RNA folding (RNA chaperone), strand exchange, and protein displacement activity (1, 6, 7). The conserved helicase core of the RNA helicases is generally flanked by variable regions which are important for determining the specificity of the enzyme (2). The cellular ability to perform these functions at low temperature is critical for cold-adapted organisms, as RNA secondary structures are stabilized at lower temperatures (8).

In bacteria, only a small number of genes (four to six) code for DEAD-box proteins (9). Gram-negative bacterium, such as *Escherichia coli*, contains five genes for different DEAD-box RNA helicases, while the Gram-positive bacterium, such as *Bacillus subtilis*, contains four DEAD-box RNA helicase genes in the genome (7, 10, 11). These helicases are involved in ribosome biogenesis, mRNA processing, and translation. Many of these proteins are non-essential in the organisms at optimum temperature of growth but are variably important for growth at low temperature. In *E. coli*, none of the five DEAD-box proteins (RhlE, SrmB, CsdA, DbpA, and RhlB) are essential for growth at 37°C (12). However, SrmB and CsdA are critical at low temperature (15°C–20°C). In contrast, RhlE, RhlB, and DbpA are dispensable for growth at low temperature (7, 8, 12, 13).

RhlE has been found associated with 70S ribosomes under normal conditions of growth. Its association to the ribosomal subunits (50S and 30S), however, has been proposed based on the analysis of the defective 40S particle in ∆*csdA*, ∆*srmB*, and ∆*csdA*∆*srmB* double mutants, suggesting that the helicase may have a direct role in the ribosome biogenesis (7, 12, 13). RhlE has been implicated in biogenesis and assembly of the 50S ribosomal subunit, where it may be involved in interconversion of two different conformational structures of 23S rRNA specific for SrmB and DbpA (13). It has been proposed that under the overexpressing conditions, RhlE favors the SrmB-specific structural conformation of 23S rRNA, while it favors the CsdA-specific conformation of 23S rRNA in the absence of RhlE. The possibility of the existence of two different conformations of 23S rRNA and their conversion explains the opposing effects of *rhlE* disruption in the ∆*srmB* and ∆*csdA* genetic backgrounds (12, 13).

SrmB has been classified as a ribosome assembly factor (8, 14 – 16). Disruption of *srmB* in *E. coli* has no associated phenotype at the optimum temperature (37°C) of growth; however, at 25°C, the ∆*srmB* mutant displays a cold-sensitive phenotype (12, 15). Molecular analysis indicated that the mutant cells exhibit a higher amount of precursor 23S rRNAs that contain unprocessed nucleotides (seven to nine bases) at 3′ ends and unprocessed 5′ ends (three to seven bases). Ribosome profiling revealed that the mutant cells contain a reduced amount of 50S subunits and an accumulation of defective 40S particles (12, 15).

DEAD-box helicase CsdA (also known as *DeaD* in *E. coli*) was found to be crucial for the growth of *E. coli* at low temperature (12). Deletion of the *csdA* gene (∆*csdA*) led not only to severe defects in growth and viability at low temperature (20°C–25°C) but also to a slow-growing phenotype at the optimum temperature (37°C). ∆*csdA* exhibited a similar type of rRNA processing and ribosome assembly defects as shown by ∆*srmB* but to a higher degree, both at the low (25°C) and optimum (37°C) temperatures of growth (12, 17, 18). The accumulation of defective ribosomal RNAs was fourfold higher in ∆*csdA* than in wild type (WT) (12). This conclusion is consistent with the growth measurements that revealed at 37°C; each of the multiple helicase deletion strain [e.g., quadruple ∆*dbpA*∆*rhlB*∆*rhlE*∆*srmB* mutants and quintuple ∆*csdA*∆*dbpA*∆*rhlB*∆*rhlE*∆*srmB* (∆5)] mutant grew more slowly than a wild-type strain, but none of them grew significantly slower than the singly mutated ∆*csdA* strain (12).

Deletion of *rhlB* and *dbpA* helicases does not produce any temperature sensitive phenotypes in *E. coli* However, a highly specific interaction of DbpA with 23S rRNA via its C-terminal domain points toward a role of the protein in ribosome biogenesis, where the helicase acts possibly by structural rearrangement of the 23S rRNA (18 – 20). Evidence for the role of DbpA in late stages of 50S subunit assembly was recently confirmed when *E. coli* strain overexpressing an inactive DbpA helicase displayed the cold-sensitive phenotype (20, 21). Some of the late ribosome binding proteins that bind close to the DbpA biding site on 23S rRNA were absent in the ribosomes (22)

In *E. coli*, the mutant strains deleted for all five DEAD-box RNA helicases in different combinations showed that the strains with multiple knockouts grow slower than the wild type at 37°C but not significantly less than the single ∆*csdA* mutant, indicating the important role of *csdA* in growth at all temperatures (12). However, at low temperature (25°C), the growth analysis revealed that there is an increase in generation time with each successive gene deletion as a result of which the strain with all five gene deletions, strain (∆5), displayed the longest generation time (181 hours) as compared to the wild type (72 hours) and even ∆*csdA* (140 hours) (12). Analysis of the rRNAs was also in conformity with fivefold more accumulation of defective rRNAs in the ∆5 strain compared to wild type. Ribosome profiling revealed a decrease in the 70S fraction and an increase in the defective 40S fraction for all cold-sensitive mutant strains (∆5, ∆*srmB*∆*csdA*, and ∆*csdA*). The defect was severe in the ∆5 strain, in which the ribosome profile was more aberrant than in ∆*srmB*, ∆*csdA*, and ∆*dbpA*, indicating the role of RhlE and RhlB helicases too in the ribosome biogenesis (12). The growth analysis of the helicase mutants in minimal media showed some interesting differences (12). At optimum temperature (37°C), there was no difference in the generation time between WT and any of the helicase mutants in the minimal growth medium; however, at 25°C, generation time of the ∆5 strain, although longer, was five times lesser compared to the wild type in rich medium (8, 12)

To understand the molecular mechanism of cold adaptation, our laboratory has been using the Antarctic bacterium *Pseudomonas syringae* Lz4W as a model organism. Draft genome sequence of *P. syringae* Lz4W has provided convincing evidence that that *P. syringae* has more sequence homology with the *Florescence* group than with the *Syringae* group of pseudomonads. These results suggest that the psychrophilic *P. syringae* should be classified as distinct species under *Pseudomona*s genus, which will be reported separately (23, 24). The major findings from our laboratory have established that the stability of DNA and RNA secondary structures have played a critical role in the adaptation and evolution of DNA and RNA metabolic enzymes, allowing bacterial growth at low temperature. Any defect in these metabolic enzymes leads to cold sensitivity of the bacterium. For example, the inactivation of the exoribonuclease enzyme RNase R, a component of the RNA degrading machinery in *P. syringae*, leads to lethality of the bacterium at cold temperature, and the bacterium fails to grow at 4°C (25). Similarly, inactivation of the DNA-repairing RecBCD machinery leads to loss of chromosomal integrity, cell lethality, and hence growth defect at low temperature (26, 27). As a follow-up of these basic findings, we have addressed the issues further by investigating the importance of the DEAD-box RNA helicases in cold adaptation, as this group of enzymes plays a major role in modulating the RNA secondary structures and RNA metabolism at low temperature.

Since DEAD-box RNA helicases act by modulating the RNA secondary structures known to be stabilized at low temperatures to regulate different aspects of RNA biology and cell physiology of cold-adapted organisms, we took a genomic approach to identify and assess the different RNA helicases that are present in the genome of the Antarctic *P. syringae* Lz4W and needed for its psychrophilic adaptation. In *P. syringae*, we identified genes for five major DEAD-box RNA helicases (*rhlE*, *srmB*, *csdA*, *dbpA*, and *rhlB*) encoding RhlE, SrmB, CsdA, DbpA, and RhlB, respectively, that are present in most Gram-negative bacteria. All of these RNA helicases contain a set of highly conserved (12) motifs including the ‘DEAD’ box (motif II) of the SF2 helicase superfamily (7, 28, 29). Since most of the studies on DEAD-box RNA helicases so far have been carried out with the above five major DEAD-box RNA helicases of mesophilic *E. coli* (12) and *Bacillus subtilis* (11), we also focused only on these five RNA helicases of the psychrophilic *P. syringae* for their role in cold adaptation.

Although the individual role of the DEAD-box RNA helicases can be inferred from the analysis of single-deletion strains, physiologically, the helicase genes work together, either co-operatively in combinations or individually by division of labor, within the cells. Sometimes, the effects of single gene inactivation might not show any phenotypic effect due to functional redundancy. Therefore, the question arises whether the cellular defects will be more pronounced if more than one helicase genes are successively deleted in different combinations, leading to either additive effects or synergistic effects, depending on their cellular interactions. To address this issue, we constructed several double-deletion strains and one triple-deletion mutant for the helicase genes in this study and assessed their growth characteristics at low (4°C) and optimum (22°C) temperatures.

The main objectives of the current study are (i) to identify and study organization of DEAD-box RNA helicase genes in *P. syringae* genome, (ii) to perform sequence analysis of RNA helicase genes including the 5′ and 3′ UTR regions, (d) to study the role of DEAD-box RNA helicases in cold-adapted growth of *P. syringae*, and (e) to identify any redundant/overlapping functions among helicases by performing functional complementation assays.

## MATERIALS AND METHODS

### Bacterial cultures and growth media

The psychrophilic *P. syringae* Lz4W was routinely grown at 22°C or 4°C (for optimum and low temperatures, respectively) in an Antarctic bacterial medium (ABM) composed of 5-g/L peptone and 2-g/L yeast extract, as described earlier (26, 30). *E. coli* strains were cultured at 37°C in Luria-Bertani (LB) medium, which contained 10-g/L tryptone, 5-g/L yeast extract, and 10-g/L NaCl (31). For solid media, 15-g/L bacto-agar (Hi Media) was added to ABM or LB. Both ABM and LB media were supplemented with ampicillin (100 µg/mL), kanamycin (50 µg/mL), spectinomycin (100 µg/mL), gentamicin (15 µg/mL), and tetracycline (10 µg/mL) as per requirement.

Generation times were calculated from the growth curves of the different recombinant strains. Fresh ABM broth was inoculated with 1% of primary culture and incubated at 22°C or 4°C with constant shaking. Optical density of bacterial culture was measured after different time intervals at 600 nm [optical density at 600 nm, OD_600_] and plotted against time.

### Molecular biology methods

General molecular biology techniques including isolation of genomic DNA, polymerase chain reactions (PCR), restriction enzyme digestion and ligation, and transformation were performed as described (31). All restriction enzymes, T4 DNA ligase and other enzymes used in this study were from New England Biolabs (USA). Polymerase chain reactions for gene amplification and site-directed mutagenesis were carried out using high-fidelity proofreading pfx DNA polymerase from Invitrogen (USA). Preparation of plasmid and purification of PCR products were done by designated Qiagen kits (Qiagen, Germany). Oligonucleotides were purchased from a commercial source (Bioserve Biotechnology, India). The conjugal transfer of recombinant plasmid into *P. syringae* was carried out by a biparental mating method using the donor *E. coli* strain S17-1. (32).

### Live/dead staining and cell viability assay

Live/dead staining of *P. syringae* cultures was performed by using LIVE/DEAD Bacterial BacLight viability kit (Invitrogen). All the steps of sample preparation and staining were performed as directed by the manufacturer (33).

### Microscopic study of *P. syringae* mutants

Cells were stained using a LIVE/DEAD BacLight bacterial viability kit (Molecular Probes) and examined under a fluorescent microscope (Axio Imager Z2, Carl Zeiss) using appropriate filters. The live/dead kit utilizes SYTO 9 green-fluorescent nucleic acid stain and the red-fluorescent nucleic acid stain propidium iodide. Wild-type and mutant strains were grown at 22°C with constant shaking until OD_600_ ~0.6 then shifted to 4°C. Cells were harvested at 22°C when OD_600_was ~0.6 and every 24 hours after shifting the culture to 4°C. Each time, 1 mL of culture was collected and centrifuged at 7,000 rpm. The pellet was washed with 0.085 M NaCl; the centrifugation step was repeated and the pellet was resuspended in 100 µL of 0.085-M NaCl. One microliter of mix dye (SYTO 9 dye and propidium iodide, 1.67 mM each) was added to cells incubated in the dark at room temperature. After 10–15 minutes of incubation at room temperature in the dark, bacterial cells were observed under a microscope at ×100 magnification.

### Construction of recombinant plasmids

All gene cloning experiments were performed in DH5α cells. The detailed methodology has been described previously (31, 34, 35). Plasmids used for the expression of RNA helicase proteins, genetic complementation of helicase mutants, and disruption of helicase genes are listed in Table 1.

**TABLE 1:** List of plasmids used in this study

Plasmid	Description	Reference or source
Plasmids for high-level expression of RNA helicases in BL21 cells		
pET28a	*Kan^r^*, expression vector for N-terminal His-tagged proteins	Novagen
pET28a*rhlE*	pET28a plasmid for overexpression of N-terminal His-tagged RHLE of *P. syringae*	This study
pET28a*srmB*	pET28a plasmid for overexpression of N-terminal His-tagged SRMB of *P. syringae*	This study
pET28a*csdA*	pET28a plasmid for overexpression of N-terminal His-tagged CSDA of *P. syringae*	This study
pET28a*dbpA*	pET28a plasmid for overexpression of N-terminal His-tagged DBPA of *P. syringae*	This study
pET28a*rhlB*	pET28a plasmid for overexpression of N-terminal His-tagged RHLB of *P. syringae*	This study
Plasmids used for functional complementation studies of RNA helicase mutants		
pGL10	Broad-host cloning vector, IncP replicon, *mob^+^*, *Kan^r^*	(36)
pGL10*rhlE*	pGL10 expressing His-tagged *P. syringae* RhlE protein	This study
pGL10*srmB*	pGL10 expressing His-tagged *P. syringae* SrmB protein	This study
pGL10*csdA*	pGL10 expressing His-tagged *P. syringae* CsdA protein	This study
pGL10*dbpA*	pGL10 expressing His-tagged *P. syringae* DbpA protein	This study
pGL10*rhlB*	pGL10 expressing His-tagged *P. syringae* RhlB protein	This study
Plasmids used as source of selective markers (antibiotic cassettes) and suicidal constructs for disruption of RNA helicase genes in *P. syringae*		
pTc28	2.4-kbp tet gene block from pOT182 cloned in pBluescript, plasmid with tetracycline resistance cassette	(26)
pKRP13	pKRP13, Amp^r^, spc/str^r^ plasmid with spectinomycin resistance cassette	(37)
pUC4K	Cloning vector, *kan ^r^* plasmid with kanamycin resistance cassette	(38)
pJQ200SK	mob^+^, *Gm^r^*, suicidal vector used for gene disruption	(39)
pJQ*rhlE-*tet	410 bp from 5′ end-tet cassette-526 bp from 3′ end of *rhlE* gene in pJQ200SK, *Gm^r^*, *tet^r^*; a suicidal construct for disruption of *rhlE* gene	This study
pJQ*srmB-kan*	422 bp from 5′ end-kan cassette-519 bp from 3′ end of *srmB* gene in pJQ200SK, *Gm^r^*, *kan^r^*, a suicidal construct for disruption of *srmB* gene	This study
pJQ*csdA-*spec	817 bp from 5′ end-spec cassette-857 bp from 3′ end of *csdA* in pJQ200SK, *Gm^r^*, *spec^r^*, a suicidal construct for disruption of *csdA* gene	This study
pJQ*dbpA-*spec	710 bp from 5′ end-spec cassette-676 bp from 3′ end of *dbpA* gene in pJQ200SK, *Gm^r^*, *spec^r^*, a suicidal construct for disruption of *dbpA* gene	This study
pJQ*rhlB-*spec	786 bp from 5′ end-*spec* cassette-705 bp from 3′ end of *rhlB* gene in pJQ200SK, *Gm^r^*, *spec^r^*, a suicidal construct for disruption of *rhlB* gene	This study

### Site-directed mutagenesis

All site-specific mutations were introduced by using the QuikChange site-directed mutagenesis kit (Agilent Technologies, USA) according to the manufacturer’s instructions. Gene-specific oligos were used for insertion of mutations in a desired gene (data not shown).

### Generation of DEAD-box RNA helicase mutant strains of *P. syringae* Lz4W

Disruption of the target gene was achieved by gene replacement method using homologous recombination between the plasmid-borne antibiotic cassette disrupted gene and the chromosomal gene as reported earlier (25, 27). A common method was followed for gene disruption, in which a selective marker (antibiotic resistance cassette) was inserted into the middle portion of the target gene cloned in suicidal plasmid pJQ200SK (26, 27, 37 – 39). For double crossover recombination to occur between the antibiotic cassette disrupted gene in the suicidal plasmid and the *P. syringae* chromosomal gene, approximately 400–500 bp of homologous DNA sequence was provided on either side of the antibiotic resistance cassette. See Fig. 6 for the schematic representation for generation of suicidal plasmid constructs used for disruption of RNA helicase genes. Numbers above the genes in the schematic refer to nucleotide numbers of the genes. Restriction sites used for cloning/insertion of antibiotic cassette are marked. The suicidal plasmid constructs generated in this study are pJQ*rhlE*-tet, pJQ*srmB*-kan, PJQ*csdA*-spec, pJQ*dbpA*-spec, and pJQ*rhlB*-spec (Table 1). Different RNA helicase mutant strains generated in this study are listed in Table 2.

**TABLE 2:** Bacterial strains used for general cloning, protein expression, and study of *P. syringae* strains disrupted for different DEAD-box RNA helicases

Bacterial strains	Genotype	Reference or source
DH5α	F− φ80*lac*ZΔM15 Δ(*lac*ZYA-*arg*F) U169 *rec*A1 *end*A1 *hsd*R17 (*r*_*k*_−, *m*_*k*_+) *pho*A *sup*E44 λ-*thi*-1 *gyr*A96 *rel*A1, used for all gene cloning purposes	(35)
S17-1	*F _ pro recA1 (r_ m_) RP4-2 integrated (Tc::Mu) (Km::Tn7) [Smr Tpr]*, used as a donor strain in conjugation	(32)
BL21 (DE3)	*F^–^ompT gal dcm lon hsdS_B_(r_B_^−^ m_B_^−^) λ[DE3 (lacI lacUV5-T7 gene 1 ind1 sam7 nin5)]*, used for overexpression of proteins under IPTG induction	(40)
*P. syringae* (Lz4W)	Lz4W Amp^r^, wild-type/natural isolate	(23)
*∆rhlE*	*rhle*::*ter^r^*, *P. syringae* strain with disrupted *rhlE* gene	This study
*∆srmB*	*srmB*::*kan^r^*, *P. syringae* strain with disrupted *srmB* gene	This study
*∆srmB∆rhlE*	*srmB*::*kan^r^*, *rhle*::*ter^r^*, *P. syringae* strain with disrupted *srmB* and *rhlE* genes	This study
*∆csdA*	*csdA*::*spec^r^*, *P. syringae* strain with disrupted *csdA* gene	This study
*∆csdA∆rhlE*	*csdA*::*spec^r^*, *rhle*::*ter^r^*, *P. syringae* strain with disrupted *csdA* and *rhlE* genes	This study
*∆dbpA*	*dbpA*::*spec^r^*, *P. syringae* strain with disrupted *dbpA* and *rhlE* genes.	This study
*∆dbpA∆rhlE*	*dbpA*::*spec^r^*, *rhle*::*ter^r^*, *P. syringae* strain with disrupted *dbpA* and *rhlE* genes	This study
*∆dbpA∆srmB*	*dbpA*::*spec^r^*, *srmB*::*kan^r^*, *P. syringae* strain with disrupted *dbpA* and *srmB* genes	This study
*∆rhlB*	*rhlB*::*spec^r^*, *P. syringae* strain with disrupted *rhlB* gene	This study
*∆rhlB∆rhlE*	*rhlB*::*spec^r^*, *rhle*::*ter^r^*, *P. syringae* strain with disrupted *rhlB* and *rhlE* genes	This study
*∆rhlB∆srmB*	*rhlB*::*spec^r^*, *srmB*::*kan^r^*, *P. syringae* strain with disrupted *rhlB* and *srmB* genes	This study
*∆rhlB∆rhlE∆srmB*	*rhlB*::*spec^r^*, *rhle*::*ter^r^ srmB*::*kan^r^*, *P. syringae* strain with disrupted *rhlB*, *rhlE* and *srmB* genes	This study

### Preparation of ɑ-^32^P labeled probe

Radiolabeled probe was prepared by using random labeling kit from JONAKI (India). About 50–100 ng of double-stranded DNA (gene specific) was denatured in a volume of 24 µL for 10 minutes in boiling water bath. The denatured DNA sample was snap frozen on ice, followed by addition of 5 µL of 10× reaction buffer, 5-µL random primer solution, 4 µL of each cold nucleotide (dCTP, dTTP, and dGTP), 2 µL of [ɑ-^32^P] dATP (hot label), and 2-µL klenow enzyme. The reaction volume was made up to 50 µL with PCR grade water and incubated at 37°C for 30–60 minutes. The hot-labeled, double-stranded DNA probe was purified from unincorporated nucleotides by passing the reaction mixture through a G-50 Sephadex chromatography column.

### Southern blotting

The DNA samples to be analyzed by Southern hybridization were digested with *Pst*I and resolved on 1% agarose gel. The gel was soaked in alkaline transfer buffer (1.0-N NaCl, 0.4-N NaOH) twice for 15 minutes. DNA from the gel was transferred on to Hybond N+ membrane by capillary transfer method using alkaline transfer buffer (1.0-N NaCl, 0.4-N NaOH) for 15–20 hours. The blot was then soaked in 0.5-M Tris-HCl (pH 7.2) and 1-M NaCl for 15 minutes and air dried. Prehybridization of the nylon membrane was carried out at 65°C for 1 hour in a prehybridization solution (0.5-M sodium phosphate buffer, 1-mM EDTA, and 7% SDS). Meanwhile, the radiolabeled double-stranded DNA probe (*rhlE* specific) was denatured in boiling water bath for 10 minutes and snap frozen on ice. The blot was then hybridized at 65°C for 15–18 hours after adding the denatured radiolabeled probe into the prehybridization solution. To reduce the background signal, the membrane was washed twice each time for 20 minutes in 20 mL of wash solution (40-mM sodium phosphate buffer, 1-mM EDTA, and 1% SDS) at 65°C. The washed membrane was covered with Saran Wrap and exposed to the photosensitive imaging plate. The plate was scanned and analyzed in phosphor imager.

### Construction of plasmids for expression and complementation studies

The helicase genes cloned in pET28-*rhlE*, pET28-*srmB*, pET28-*csdA*, pET28-*dbpA*, and pET28-*rhlB* (Table 1) were released along with the ribosome-binding site of pET28a with XbaI and SacI restriction enzymes, and cloned into the *XbaI*/*SacI* sites of broad host range plasmid pGL10 (36, 40). The resultant plasmids pG*rhlE*, pG*srmB*, pG*csdA*, pG*dbpA*, and pG*rhlB* (Table 1) harboring the respective genes for RhlE, SrmB, CsdA, DbpA, and RhlB helicases were propagated in *E. coli* DH5α and later transformed into *E. coli* S17-1 for their mobilization into *P. syringae* strains. The plasmids pGL10, pG*rhlE*, pG*srmB*, pG*csdA*, pG*dbpA*, and pG*rhlB* were mobilized separately into each individual RNA helicase mutant of *P. syringae* by conjugation with *E. coli* S17-1 (32). All the strains used in the genetic complementation study are listed in Table 3.

**TABLE 3:** *P. syringae* strains used in functional complementation study of RNA helicase mutants

Plasmid	Description	Reference/source
Lz4W (pGL10)	Wild-type strain complemented by empty pGL10 plasmid	This study
Lz4W (pGL10*rhlE*)	Wild-type strain complemented by *rhlE*	This study
Lz4W (pGL10s*rmB*)	Wild-type strain complemented by *srmB*	This study
Lz4W (pGL10*csdA*)	Wild-type strain complemented by *csdA*	This study
Lz4W(pGL10*dbpA*)	Wild-type strain complemented by *dbpA*	This study
Lz4W (pGL10*rhlB*)	Wild-type strain complemented by *rhlB*	This study
*∆rhlE* (pGL10)	*∆rhlE* strain complemented by empty pGL10 plasmid	This study
*∆rhlE* (pGL10*rhlE*)	*∆rhlE* strain complemented by *rhlE*	This study
*∆rhlE* (pGL10*srmB*)	*∆rhlE* strain complemented by *srmB*	This study
*∆rhlE* (pGL10*csdA*)	*∆rhlE* strain complemented by *csdA*	This study
*∆rhlE* (pGL10*dbpA*)	*∆rhlE* strain complemented by *dbpA*	This study
*∆rhlE* (pGL10*rhlB*)	*∆rhlE* strain complemented by *rhlB*	This study
*∆srmB* (pGL10)	*∆srmB* strain complemented by empty pGL10 plasmid	This study
*∆srmB* (pGL10*rhlE*)	*∆srmB* strain complemented by *rhlE*	This study
*∆srmB* (pGL10*srmB*)	*∆srmB* strain complemented by *srmB*	This study
*∆srmB* (pGL10*csdA*)	*∆srmB* strain complemented by *csdA*	This study
*∆srmB* (pGL10*dbpA*)	*∆srmB* strain complemented by *dbpA*	This study
*∆srmB* (pGL10*rhlB*)	*∆srmB* strain complemented by *rhlB*	This study
*∆csdA* (pGL10)	*∆csdA* strain complemented by empty pGL10 plasmid	This study
*∆csdA* (pGL10*rhlE*)	*∆csdA* strain complemented by *rhlE*.	This study
*∆csdA* (pGL10*srmB*)	*∆csdA* strain complemented by *srmB*.	This study
*∆csdA* (pGL10*csdA*)	*∆csdA* strain complemented by *csdA*	This study
*∆csdA* (pGL10*dbpA*)	*∆csdA* strain complemented by *dbpA*	This study
*∆csdA* (pGL10*rhlB*)	*∆csdA* strain complemented by *rhlB*.	This study
*∆dbpA* (pGL10)	*∆dbpA* strain complemented by empty pGL10 plasmid	This study
*∆dbpA* (pGL10*rhlE*)	*∆dbpA* strain complemented by *rhlE*	This study
*∆dbpA* (pGL10*srmb*)	*∆dbpA* strain complemented by *srmb*	This study
*∆dbpA* (pGL10*csdA*)	*∆dbpA* strain complemented by *csdA*	This study
*∆dbpA* (pGL10*dbpA*)	*∆dbpA* strain complemented by *dbpA*	This study
*∆dbpA* (pGL10*rhlB*)	*∆dbpA* strain complemented by *rhlB*	This study
*∆rhlB* (pGL10)	*∆rhlB* strain complemented by empty pGL10 plasmid	This study
*∆rhlB* (pGL10*rhlE*)	*∆rhlB* strain complemented by *rhlE*	This study
*∆rhlB* (pGL10*rhlE*)	*∆rhlB* strain complemented by *rhlE*	This study
*∆rhlB* (pGL10*srmB*)	*∆rhlB* strain complemented by *srmB*	This study
*∆rhlB* (pGL10*csdA*)	*∆rhlB* strain complemented by *csdA*	This study
*∆rhlB* (pGL10*dbpA*)	*∆rhlB* strain complemented by *dbpA*	This study
*∆rhlB* (pGL10*rhlB*)	*∆rhlB* strain complemented by *rhlB*	This study

## RESULTS

### Genome organization of the DEAD-box RNA helicase genes in *P. syringae*

**Fig 1:**
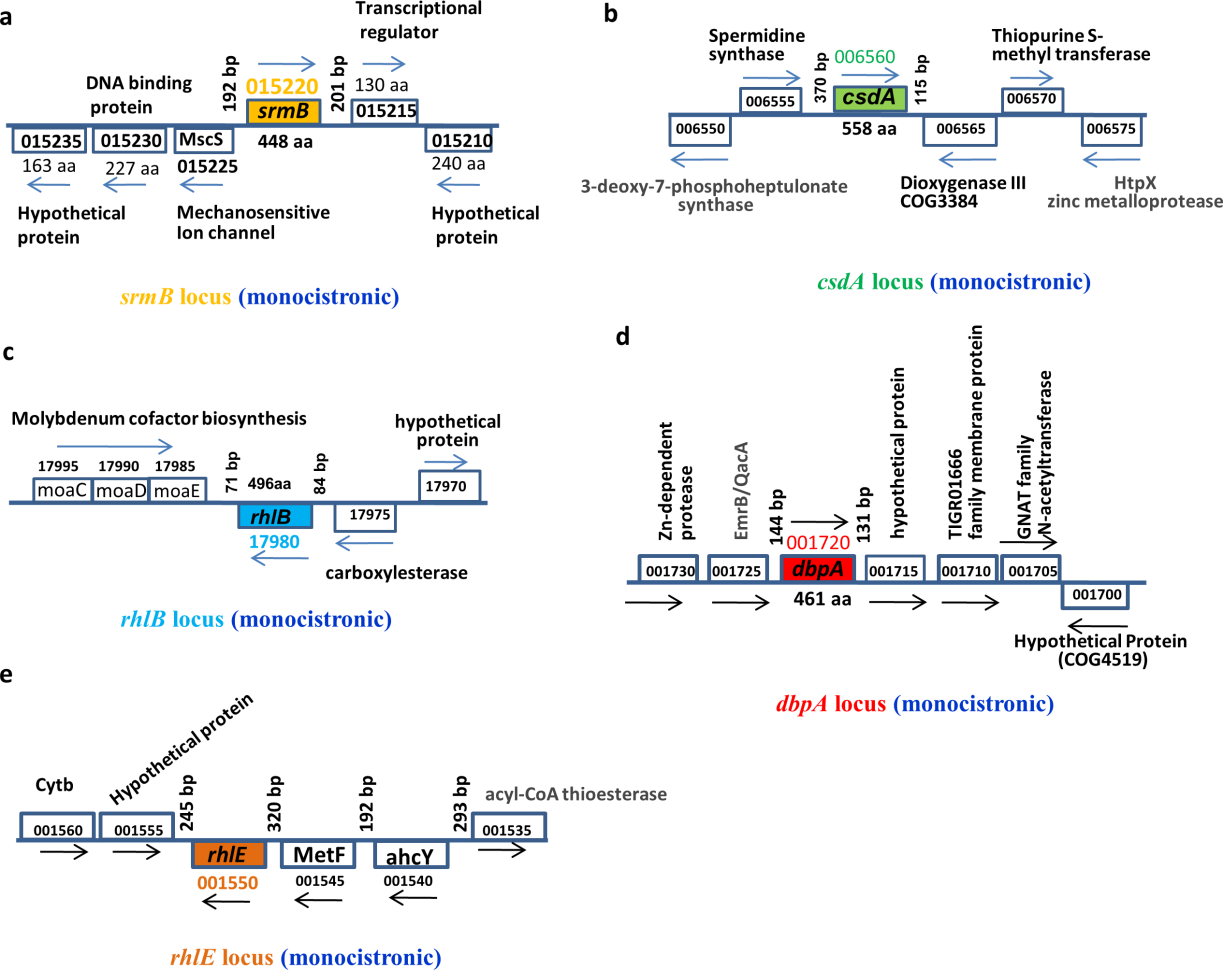
Genomic organization of the five DEAD-box RNA helicase genes of *P. syringae* Lz4W. The helicase genes and their surrounding upstream and downstream genes have been indicated by their respective locus tag numbers in the *P. syringae* genome. The locus tags in the annotated genome (https://www.ncbi.nlm.nih.gov/nuccore/CP017432.1/accession number: AOGS01000001-AOGS01000042) at the National Center for Biotechnology Information site starts with locus tag number B195_000005 to B195_022465. For clarity on the figure, the common prefix “B195_” has been omitted in the diagram. The direction of transcription is shown by arrows on above or below the gene-ORF boxes. The encoded gene product lengths are shown by amino acid numbers. (a) *srmB* gene locus (b) *csdA* gene locus, (c) *rhlB* locus, (d) *dbpA* gene locus, and (e) *rhlE* gene locus.

Analysis of the *P. syringae* Lz4W genome sequence helped us to identify five DEAD-box RNA helicase genes in the chromosome of the bacterium. We identified the genes (*rhlE*, *srmB*, *csdA*, *dbpA*, and *rhlB*) for the five major DEAD-box RNA helicases of Gram-negative bacteria which encoded RhlE, SrmB, CsdA, DbpA, and RhlB, respectively. The organization of DEAD-box RNA helicase genes, namely, *rhlE*, *srmB*, *csdA*, *dbpA*, and *rhlB*, encoded by genetic loci, B195_001550, B195_015220, B195_006560, B195_001720, and B195_17980, respectively, on the *P. syringae* genome is shown in Fig. 1. From the analysis of directions of transcription and the distance between the genes, we inferred that these RNA helicase genes are monocistronic and dispersed over different parts of the *P. syringae* genome.

**Fig 2:**
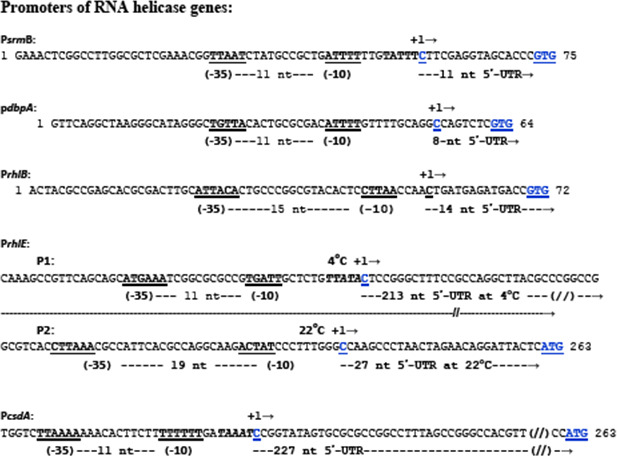
Putative regulatory regions of *P. syringae* helicase genes showing promoter characteristics and 5′-UTR lengths of the five major DEAD-box RNA helicase genes of *P. syringae* Lz4W. The “−10” and “−35” sequences of the promoters have been underlined at the upstream of (+1) transcription-start sites that are all located “ATG” or “GTG” translation initiation codons of the respective genes. The lengths of the 5′-UTR’s have been indicated for each of the transcripts of the helicase genes. To note that *rhlE* helicase genes have two promoters, P1 and P2. The transcript from the P1 promoter is observed at low temperature (4°C), and that from the P2 promoter is produced at optimum temperature (22°C) of growth. The transcript start sites were based on transcriptome data of the laboratory (M. K. Ray, unpublished data).

**Fig 3:**
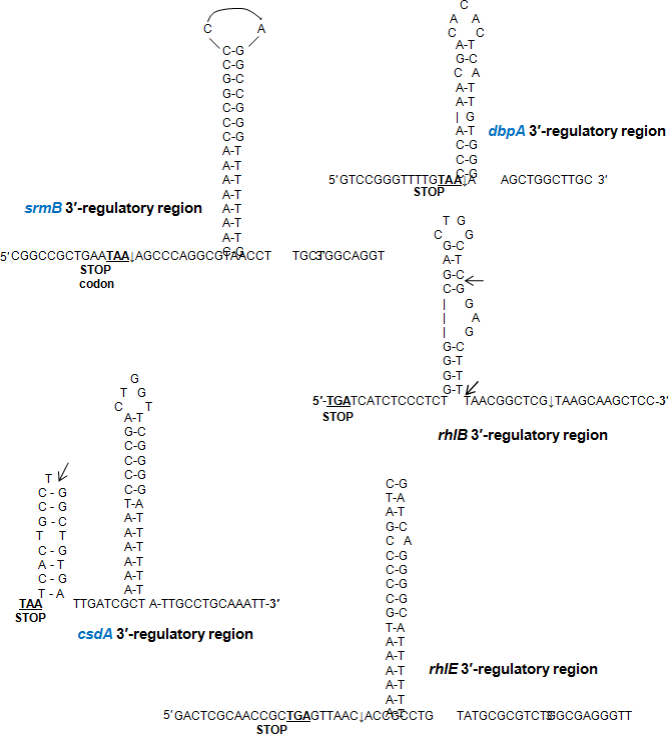
Regulatory 3′ regions of *P. syringae* helicase genes showing the putative 3′ end hairpin structures for transcription termination. The translational “stop” codon of the genes are underlined. The arrows indicate the transcription stop site, that were determined in a separate study in the laboratory (M. K. Ray, unpublished data).

The upstream and downstream intergenic spacers contained the putative promoter and regulatory sequences of the helicase genes which were of variable lengths (Fig. 2). The *rhlB* upstream and downstream intergenic spacers had the shortest lengths corresponding to 84 and 71 bp sequences, respectively. The *csdA* had the longest upstream intergenic spacer spanning 370 bp sequences. The *srmB* and *dbpA* genes had modest upstream intergenic spacers of 192 and 144 bp, respectively. The lengths of the downstream intergenic distances varied, each housing putative transcription terminators for the respective gene (Fig. 3).

### Variation among *P. syringae* DEAD-box RNA helicases and their similarities to the homologs from other *Pseudomonas* species

**Fig 4:**
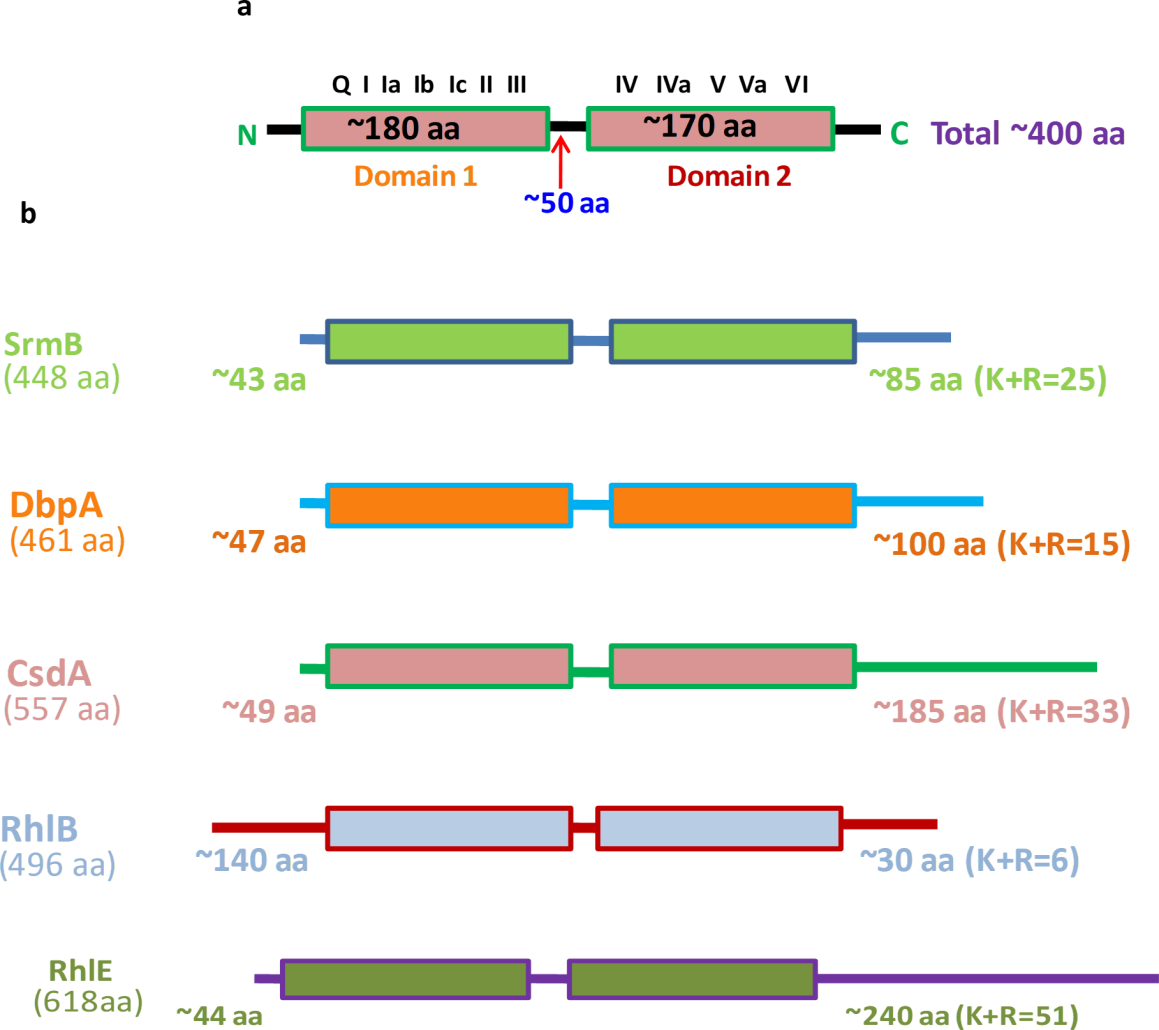
Domain organization of DEAD-box RNA helicases. (a) Schematic representation of a typical RNA helicase showing twin domain catalytic center and linker. The domains are flanked by variable N and C terminal regions. Also shown in the picture are different and highly conserved motifs involved in ATP hydrolysis and unwinding activities, whereas the terminal regions are involved in binding substrate. (b) Diagram representing number of amino acids, N and C terminal variable extensions, and K + R of C-terminal extension in all five DEAD box RNA helicases of *Pseudomonas syringae*.

The five major DEAD-box RNA helicases, *rhlE*, *srmB*, *csdA*, *dbpA*, and *rhlB*, encode the proteins of different sizes, i.e., RhlE (618 residues), SrmB (448 residues), CsdA (557 residues), DbpA (461 residues), and RhlB (496 residues), corresponding to molecular mass of approximately 68, 49, 61, 50, and 54 kDa, respectively. They all displayed the conserved domain organization typical for the DEAD-box RNA helicases found in different organisms. (Fig. 4a). The highly conserved catalytic helicase core comprising the D1 and D2 domains and variable N- and C-terminal extensions of the *P. syringae* RNA helicase proteins are shown schematically in Fig. 4b. The N-terminal extensions are about 43–49 amino acids long except for the RhlB, which showed an extension of ~140 amino acids. The C-terminal extensions were also of different lengths containing a variable number of positively charged amino acid residues, which might have a bearing on interaction of the proteins with the negatively charged RNA substrates. The catalytic core of the RNA helicase contains 12 conserved motifs that are responsible for ATPase and helicase activities of the protein.

**Fig 5:**
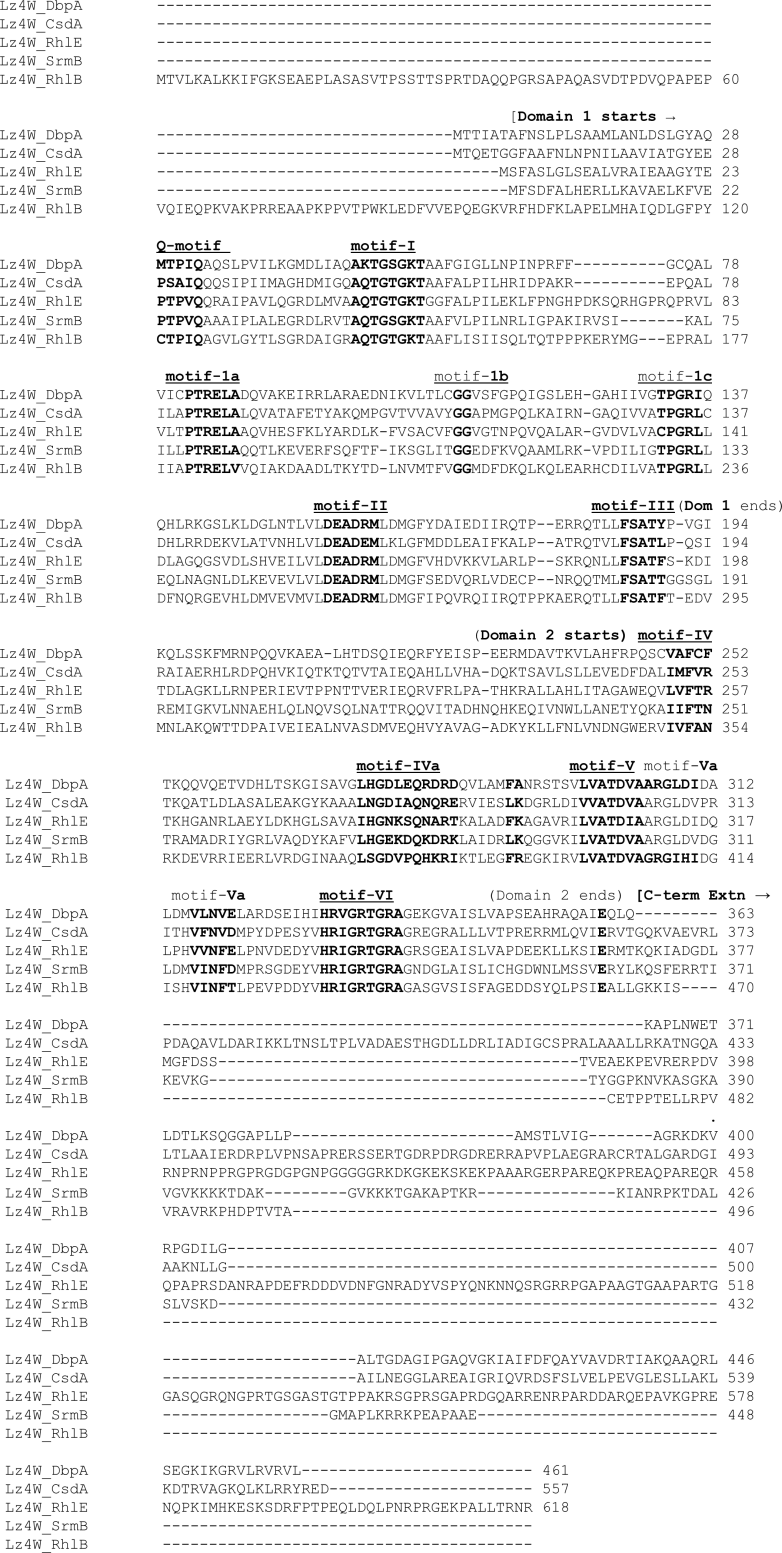
Multiple sequence alignment-based analysis of DEAD-box RNA helicases. Conserved structural motifs of the RNA helicases shown above the aligned amino acid sequences of the five major DEAD box RNA helicases encoded by the *P. syringae* Lz4W genome. The beginning and end of the two structural domains with C-terminal extensions of the RNA helicases have also been marked. The multiple sequence alignment-based analysis was performed by ClustalW. The locus tags of the RNA helicase genes and their respective protein IDs are as follows: *dbpA* locus tag B195_001720, protein id AUB73597.1; *csdA* locus tag B195_006560, protein id AUB74496.1; *rhlE* locus tag B195_001550, protein id AUB73564.1; *srmB* locus tag B195_015220, protein id AUB76136.1; and *rhlB* locus tag B195_017980, protein id AUB76657.1 (GenBank: CP017432.1, National Center for Biotechnology Information).

Multiple sequence alignments of the five RNA helicase proteins of *P. syringae* Lz4W suggested that the helicases were about 32%–39% identical among themselves in the amino acid sequences, with the lowest identity (32%) observed between CsdA and SrmB and the highest (39%) shown by RhlB and RhlE (Fig. 5 and Table 4). An analysis with the *E. coli* RNA helicase homologs yielded similar results, except that the DbpA and CsdA helicases too, like the RhlB and RhlE, exhibited 39% identity. Thus, all the DEAD-box RNA helicases might have their common ancestry from which SrmB helicase might have diverged early in the evolution. In general, the respective *P. syringae* DEAD-box helicases show about 70%–88% identity with the *P. aeruginosa* homologs and 36%–57% identity with the *E. coli* homologs in the amino acid sequences (Table 5). Thus, the *Pseudomonas* homologs of the RNA helicases have diverged substantially from the *E. coli* helicases while retaining similar cellular functions.

**TABLE 4:** Identity among the six RNA helicases that are encoded by *P. syringae* Lz4W

Sequences	RNA helicase	Number of amino acids		DbpA	CsdA	RhlE	SrmB	RhlB
1	Lz4W_DbpA	461	Lz4W_DbpA	100.0	36.66	32.75	33.58	35.42
2	Lz4W_CsdA	551	Lz4W_CsdA	36.66	100.0	32.62	32.65	34.26
3	Lz4W_RhlE	618	Lz4W_RhlE	32.75	32.62	100.0	36.32	39.14
5	Lz4W_SrmB	448	Lz4W_SrmB	33.58	32.65	36.32	100.0	35.57
6	Lz4W_RhlB	496	Lz4W_RhlB	35.42	34.26	39.14	35.57	100

**TABLE 5:** Percentage identity of DEAD box RNA helicases from the psychrophilic *P. syringae* Lz4W, and mesophilic *P. aeruginosa* and *Escherichia coli*

*P. syringae* RNA helicase	Identity (%) with mesophilic homologues	
	*P. aeruginosa*	*E. coli*
1. CsdA (618 aa)	(567 aa); 75.86%	(629 aa); 46.47%
2. SrmB (448 aa)	(446 aa); 73.24%	(444 aa); 36.9%
3. DbpA (461 aa)	(354 aa); 77.68%	(457 aa); 56.04%
4. RhlB (496 aa)4. RhlB (496 aa)	(397 aa); 88.16%	(421 aa); 42.36%
5. RhlE-Large (618 aa) [degradosome associated]	(639 aa); 70.18%	(454 aa); 57.96%

### Importance of DEAD-box RNA helicase gene deletions in cold-adapted growth of *P. syringae*

**Fig 6:**
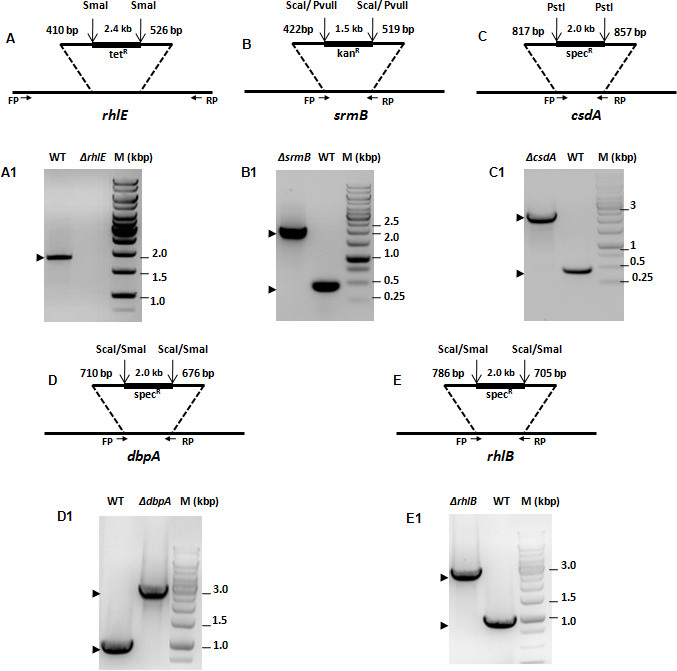
Generation and validation of RNA helicase mutants in *P. syringae*. Schematic representation of suicidal plasmid constructs employed for disruption of RNA helicase genes in wild type (WT) genetic background. Numbers above the gene in the schematic refer to nucleotide number of the plasmid-borne gene. Restriction sites used for insertion of selective markers are indicated. The sizes of the selective markers are also mentioned. (A) Schematic representation of suicidal plasmid construct pJQ*rhlE-*tet used for disruption of the *rhlE* gene. (A1) Lanes marked as WT and ∆*rhlE* represent the PCR amplification results of strain-specific genomic DNA with *rhlE* specific primers designed from each end of the *rhlE* gene. The disruption of *rhlE* gene was also confirmed by Southern blotting (Fig. 7) (B) Schematic representation of suicidal plasmid construct pJQ*srmB-*kan used for disruption of the *srmB* gene. (B1) Lanes marked as WT and ∆*srmB* represent the PCR amplification results of strain-specific genomic DNA with *srmB*-specific primers designed to amplify 225 bp from each end of the inserted kan^r^ cassette. (C) Schematic representation of suicidal plasmid construct pJQ*csdA-*spec used for disruption of the *csdA* gene. (C1) Lanes marked as WT and ∆*csdA* represent the PCR amplification results of strain specific genomic DNA with *csdA* specific primers designed to amplify 250 bp from each end of spec^r^ cassette. (D) Schematic representation of suicidal plasmid construct pJQ*dbpA-*spec used for disruption of the *dbpA* gene. (D1) Lanes marked as WT and ∆*dbpA* represent the PCR amplification results of strain-specific genomic DNA with *dbpA* specific primers designed to amplify 500 bp from each end of inserted spec^r^ cassette. (E) Schematic representation of suicidal plasmid construct pJQ*rhlB-*spec used for deletion of the *rhlB* gene. (E1) Lanes marked as WT and ∆*rhlB* represent the PCR amplification results of strain specific genomic DNA with *rhlB* specific primers designed to amplify 500 bp from each end of the inserted spec^r^ cassette.

**Fig 7:**
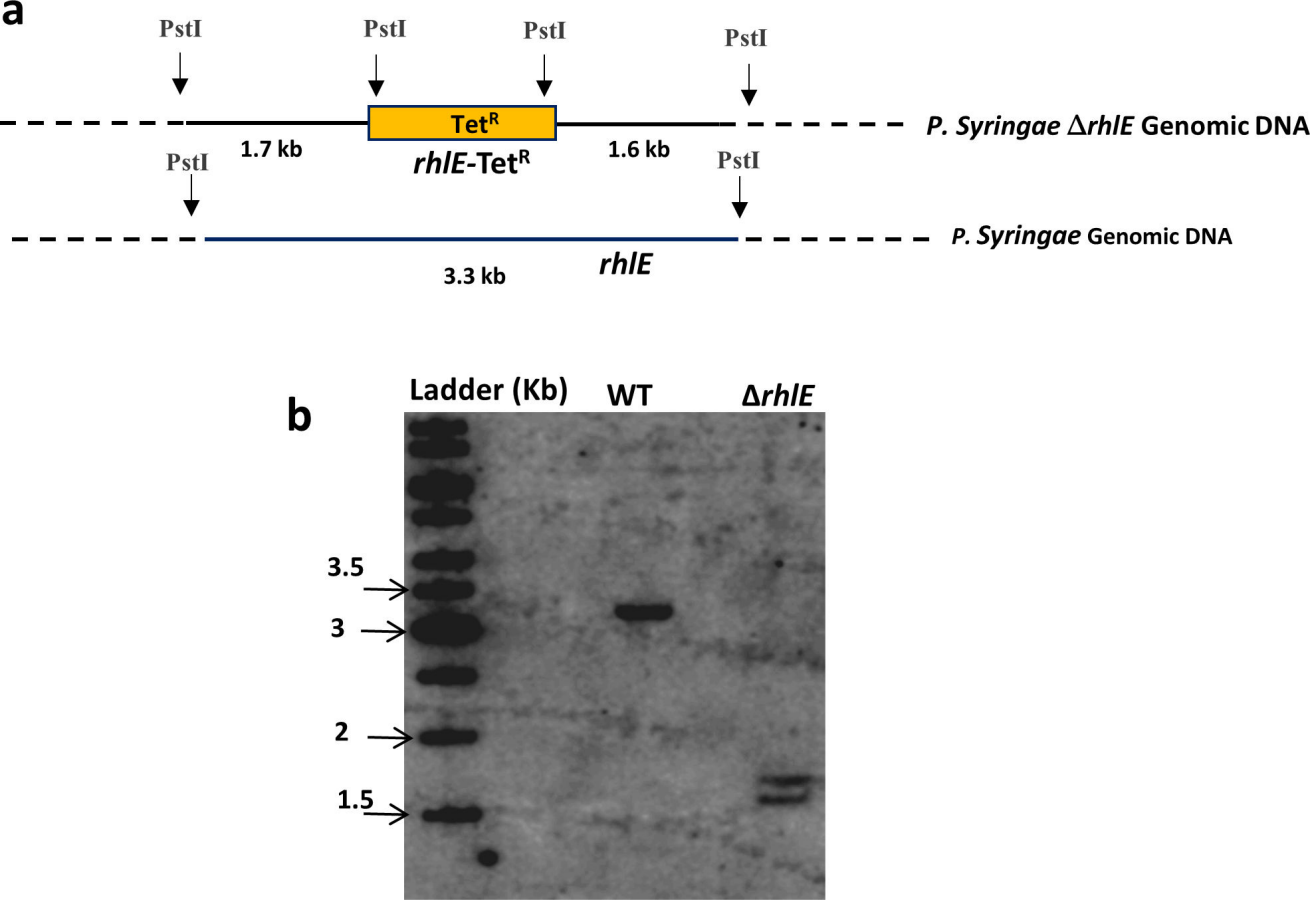
Southern blot analysis of sole *rhlE* mutant. (a). Schematic representation of the *rhlE* gene and associated PstI sites in genome of *Pseudomonas syringae* Lz4W and Tetracycline cassette mediated disrupted *rhlE* gene in wild type genetic background. (b) Verifying *rhlE* mutant using southern blot: Disruption of *rhlE* gene was also confirmed by Southern hybridization method, in which PstI digested genomic DNAs from wild-type and ∆*rhlE* was transferred to nylon membrane and probed with ^32^P-labeled *rhlE* DNA. In the Southern analysis, WT sample produced one hybridization band corresponding to a size of 3.3 kb, whereas the ∆*rhlE* sample produced two hybridization signals corresponding to 1.6 and 1.7 kbp DNA bands. Size of hybridized band in wild-type lane equals to the sum of two bands in ∆*rhlE* lane, confirming that PstI cleaves the *rhlE* DNA fragment approximately in the middle, which is in well agreement with the PstI sites located at both ends of the Tet^R^ cassette, enabling removal of tetracycline cassette from genomic *rhlE*::Tet^r^ DNA of mutant strain.

**Fig 8:**
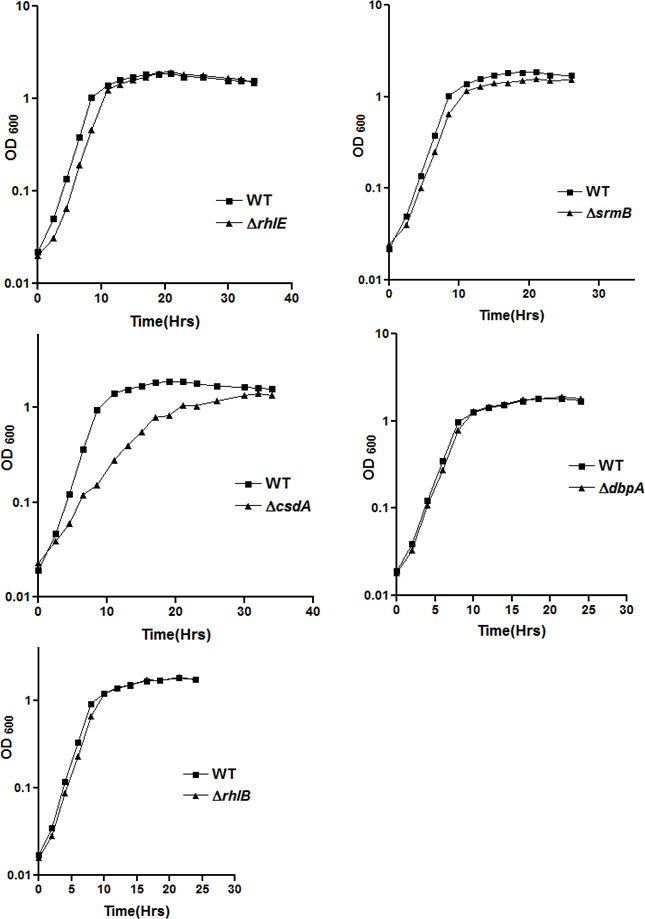
Growth analysis of *P. syringae* helicase mutants ∆*rhlE* (a), ∆*srmB* (b), ∆*csdA* (c), ∆*dbpA* (d) and ∆*rhlB* (e) at 22°C. For measurement of growth, mutant strains of *P. syringae* were grown separately in ABM broth at 22°C and OD at 600nm [OD_600_] was recorded at regular time intervals and plotted against time. Each growth curve has been performed at least three times. All growth curves were generated using GraphPad Prism 4.0 software.

**Fig 9:**
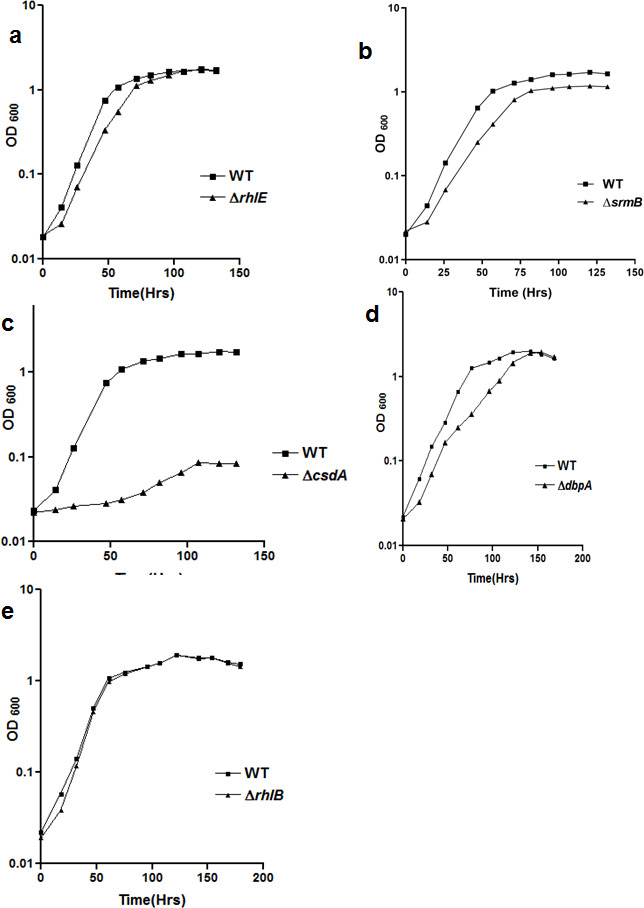
Growth analysis of *P. syringae* helicase mutants ∆*rhlE* (a), ∆*srmB* (b), ∆*csdA* (c), ∆*dbpA* (d) and ∆*rhlB* (e) at 4°C. For measurement of growth, mutant strains of *P. syringae* were grown separately in ABM broth at 4°C and OD at 600nm [OD_600_] was recorded at regular time intervals and plotted against time. Each growth curve has been performed at least three times All growth curves were generated using GraphPad Prism 4.0 software

To decipher the role of the DEAD-box RNA helicase genes in low-temperature adapted growth of *P. syringae*, mutant strains were constructed for each helicase gene by gene replacement through homologous recombination (Fig. 6 and Tables 1 and 2). The disruption of *rhlE* gene was also confirmed by Southern blotting (Fig. 7). Growth analysis of mutant strains at 22°C revealed that ∆*rhlE*, ∆*srmB*, ∆*dbpA*, and ∆*rhlB* strains displayed a growth phenomenon similar to that of the WT, whereas growth of the ∆*csdA* strain was slightly impaired (Fig. 8). Measurement of generation times confirmed that ∆*rhlE*, ∆*srmB*, ∆*dbpA*, and ∆*rhlB* have the same growth rates as of wild type; however, the generation time of ∆*csdA* (3.97 hours) is almost twice that of wild type (2.02 hours) (Table 6). The growth study of all five mutants at low temperature (4°C) divulged interesting results. Helicase mutants ∆*rhlE* and ∆*rhlB* showed growth characteristics like wild type; however mutants ∆*srmB* and ∆*dbpA* displayed impaired growth, whereas ∆*csdA* mutant displaying a completely cold-sensitive phenotype did not exhibited any measurable growth (Fig. 9). Measurement of generation times confirmed that ∆*rhlE* and ∆*rhlB* have the same growth rate as that of wild type; however, the generation time of ∆*srmB* (13.0 hrs) and ∆*dbpA* (11.9 hrs) is significantly longer than that of wild-type strain (6.75 hrs). The results of growth analysis for *P. syringae* strains ∆*csdA* (22°C and 4°C) and ∆*dbpA* (4°C) were different from the results obtained with mesophilic *E. coli*, where ∆*csdA* only exhibits a slow-growing phenotype at low temperature (25°C). These findings indicate that helicase genes *srmB* and *dbpA* are important, but *csdA* is indispensable for growth of bacterium at low temperature (4°C).

**TABLE 6:** Effects of helicase mutations on cell viability [in 4°C shifted cultures] and generation time of mutant strains grown at 22°C and 4°C^*b*^

Strains	Dead cells (%) at 4°C			Generation time (hours)	
	24 hrs	48 hrs	72 hrs	At 22°C	At 4°C
WT	0.2 ± 0.11	1.5 ± 0.08	1.4 ± 0.2	2.02 ± 0.21	6.75 ± 0.5
∆*csdA*	41.0 ± 5.02	50.5 ± 4.9	52.2 ± 1.6	3.97 ± 0.84	No growth^*a*^
∆*srmB*	21.8 ± 0.25	29.8 ± 0.75	48.3 ± 0.9	2.03 ± 0.14	13.0 ± 1.2
∆*dbpA*	6.6 ± 0.23	4.8 ± 0.4	3.2 ± 0.2	2.4 ± 0.47	11.9 ± 0.7
∆*rhlB*	0.5 ± 0.14	2.6 ± 0.17	3.2 ± 0.2	2.07 ± 0.17	8.6 ± 0.9
∆*rhlE*	1.8 ± 0.2	4.0 ± 0.6	2.2 ± 0.1	2.1 ± 0.13	7.4 ± 1.6

^*a*^ *csdA* disrupted mutants do not display growth at 4°C.

^*b*^ Dead cells were scored by counting the PI-stained cells (red) and expressed as the % of total cells in the microscopic fields from a minimum of three different areas.

The growth phenotypes of RNA helicase mutant strains grown at 22°C or 4°C (after shift from 22°C) were further investigated by cell viability assays using live/dead staining of the cells in growing cultures. After 72 hours of incubation at 4°C, approximately 52% of ∆*csdA* cells and 48% of ∆*srmB* cells were stained red (dead), as compared to wild type, where only 1% of cells were stained red (Table 6). The results obtained with live/dead staining of wild-type and mutant strains are well in agreement with the results obtained from the growth curve analysis of desired mutant strains. The mutant strains with increased generation time at low temperatures displayed high percentage of cell death at low temperatures. The anomaly was with the ∆*dbpA* strain, which displayed a prolonged generation time (11.9 hours) similar to that of the ∆*srmB* strain (12.9 hours); however, the percentage of dead cells was very less than expected. The low percentage of cell death may be due to better acclimatization of ∆*dbpA* to low temperature (4°C) after the shift from optimal conditions of growth (22°C).

**Fig 10:**
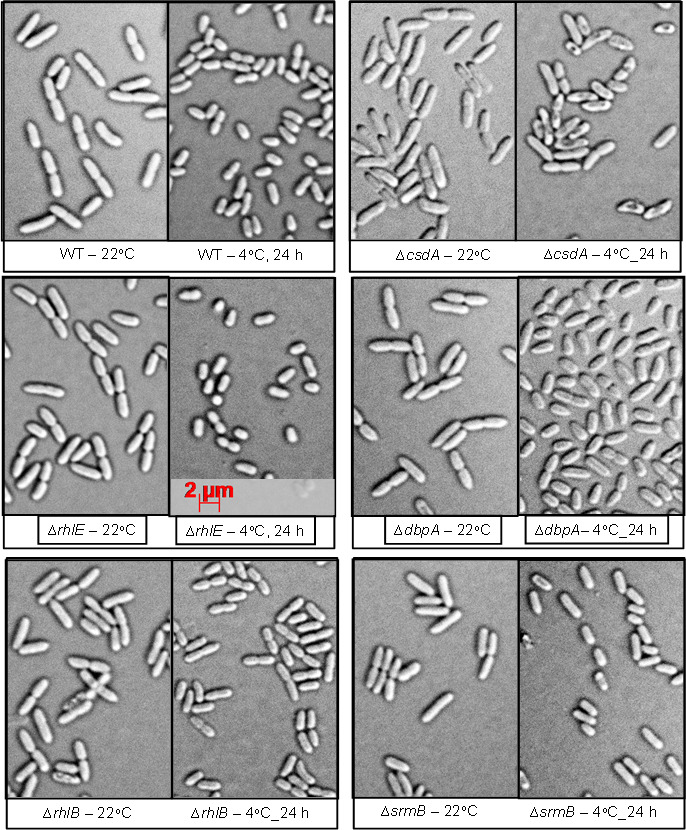
Morphological changes in RNA helicase mutants of *P. syringae* at 22°C and 4°C. For microscopic study of bacterial cells, wild type and mutant strains were grown at 22°C with constant shaking till OD at 600 nm reaches 0.6, and then shifted to 4°C. Cells were harvested at 22°C when OD at 600 nm reaches 0.6 and every 24 hrs after shifting the cultures to 4°C. Each time 1 ml of bacterial culture was collected and centrifuged at 7000 rpm. The pellet was washed with 0.085M NaCl, centrifugation step was repeated and pellet was resuspended in 100 µl of 0.085M NaCl. After 5 minutes of incubation at room temperature cells were observed under microscope (Carl Zeiss, Axio imager Z2) at 100X magnification. The DIC images were captured for morphological studies of bacterial cells like analysis of shape, size and general appearance.

Microscopic studies of helicase disrupted strains revealed a decrease in the cell size as one of the major effects of exposure to low temperature (4°C), which has also been reported earlier (25, 26). Like WT cells, ∆*rhlE* and ∆*rhlB* strains did not display any low temperature-associated change in morphology (Fig. 10). ∆*csdA* mutants grown at 22°C displayed a dull appearance, whereas ∆*csdA* cells grown at 4°C displayed a speck of dark materials at the middle of the lateral along the long axis, giving an appearance of puncture at the center of the cells. Helicase mutants ∆*srmB* and ∆*dbpA* did not exhibit any changes in appearance at 22°C; however, when these mutants were grown at 4°C, the mutants revealed a dull appearance and were fractionally elongated and bigger in size than wild-type cells growing at the same (4°C) temperature. The low temperature-associated morphological changes observed with ∆*srmB*, ∆*csdA*, and ∆*dbpA* mutants might be an indirect effect of *srmB*, *csdA*, or *dbpA* gene disruption in mutant cells, or these genes might have an indirect role in the modulation of cell shape and size in *P. syringae* especially at low temperature. The magnitude of low temperature-associated changes in morphology with ∆*srmB* and ∆*dbpA* mutants was less pronounced as compared to the effects seen with ∆*csdA*.

### Growth phenotypes of the double and triple helicase deletion strains

**Fig 11:**
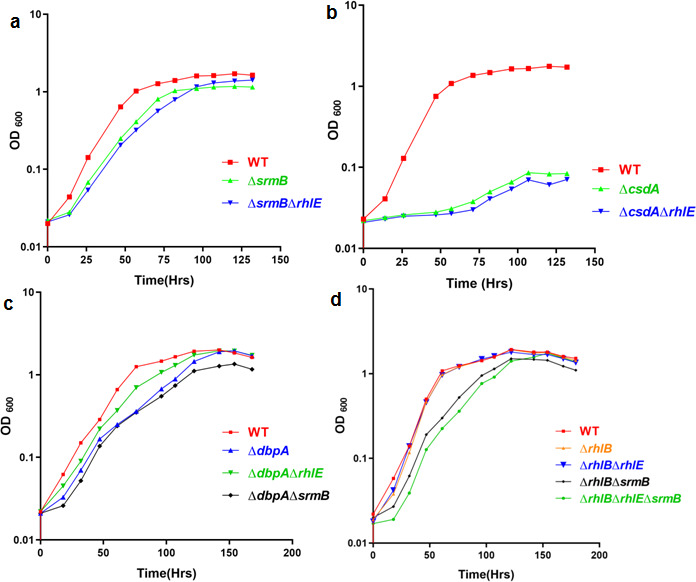
Growth analysis of DEAD-box RNA helicase mutants harboring multiple disruptions. Growth analysis of *srmB* (a) and *csdA* (b) gene disruptions in different mutant backgrounds was performed at 4°C. Growth study of *∆dbpA* (*∆dbpA∆rhlE*) and (*∆dbpA∆srmB*) strains at 4°C is shown in (c). Similarly, effects of *rhlB* deletion in various helicase mutant backgrounds were analyzed by comparing the growth profiles of *∆rhlB* (*∆rhlB ∆rhlE*), (*∆rhlB ∆srmB*), and (*∆rhlB ∆rhlE ∆srmB*) strains at 4°C (d). For measurement of growth, different strains of *P. syringae* were grown separately in ABM broth at 4°C. OD at 600 nm (600_nm_) was recorded at regular intervals and plotted against time. Each growth curve was generated at least three times. All growth curves were generated using GraphPad Prism v.4.0 software.

The disruption of *rhlE* gene in the ∆*srmB* background generating the double mutant (∆*rhlE*∆*srmB*) did not lead to any rescue of the slow-growing phenotype in the ∆*srmB* mutant of *P. syringae*, as observed in the case of *E. coli* (12). There was no difference in the growth profiles between ∆*srmB* and ∆*srmB*∆*rhlE* at low temperature (Fig. 11a). This suggested that the *P. syringae* RhlE helicase does not probably interact with the SrmB-depleted 50S ribosomal subunit, as observed in *E. coli*, where RhlE was proposed to modulate the interconversion of two alternative forms of 50S ribosomal subunit intermediates. SrmB requirement (possibly in the ribosome assembly) during the growth of psychrophilic *P. syringae* at low temperature is independent of R.

**Fig 12:**
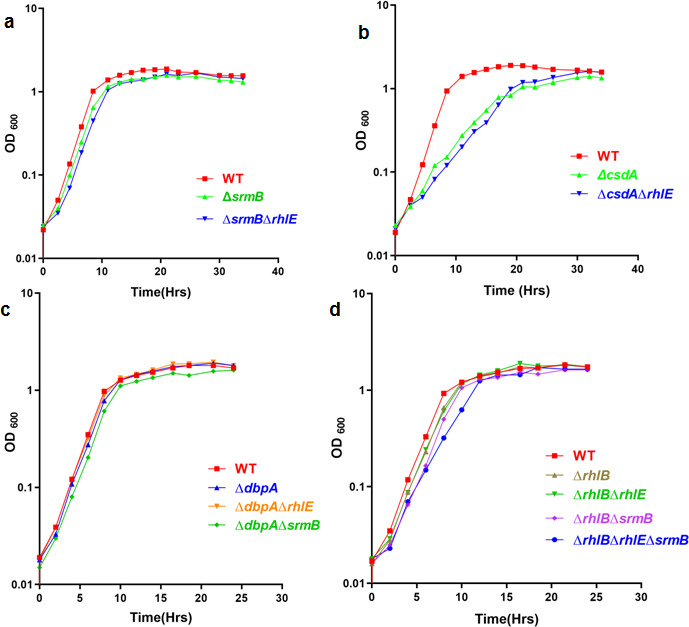
Growth analysis of DEAD box RNA helicase mutants harbouring multiple disruptions. Growth analysis of *srmB* (a) and *csdA* (b) gene disruptions in different mutant backgrounds was performed at 22°C. Growth of *∆dbpA*, (*∆dbpA∆rhlE*) and (*∆dbpA∆srmB*) strains at 22°C are shown in (c). Similarly, effects of *rhlB* deletion in various helicase mutant backgrounds was analyzed by comparing the growth profiles of *∆rhlB*, (*∆rhlB ∆rhlE*), (*∆rhlB ∆srmB*), and (*∆rhlB ∆rhlE ∆srmB*) strains at 22°C (d). For measurement of growth, different strains of *P. syringae* were grown separately in ABM broth at 22°C. OD at 600nm [600_nm_] was recorded at regular intervals and plotted against time. Each growth curve has been performed at least three times. All growth curves were generated using GraphPad Prism 4.0 software.

hlE function was mainly mediated through RNA degradosome in the bacterium. Similarly, but in contrast to *E. coli*, both ∆*csdA* and ∆*csdA*∆*rhlE* double mutant of *P. syringae* displayed no alteration in the cold-sensitive phenotype of the ∆*csdA* mutant (Fig. 11b). In *E. coli*, deletion of the *csdA* gene in ∆*rhlE* background exacerbated the cold-sensitive phenotype of ∆*csdA*.

Disruption of the *dbpA* gene in wild-type, ∆*rhlE*, and ∆*srmB* genetic backgrounds has some interesting effects on the growth and viability of the strains. Growth of ∆*dbpA*, ∆*dbpA*∆*rhlE*, and ∆*dbpA*∆*srmB* strains at 22°C and 4°C is shown in (Fig 11c and 12c). All strains including the wild-type, ∆*dbpA*, ∆*dbpA*∆*rhlE* and ∆*dbpA*∆*srmB* displayed normal and similar growth at 22°C (Fig. 12). However, at 4°C, while ∆*dbpA* displayed a marginally cold-sensitive phenotype, the *dbpA* deletion in ∆*rhlE* background ameliorated the cold-sensitive phenotype of ∆*dbpA* strain. The ∆*dbpA*∆*rhlE* double mutant grew better than the single ∆*dbpA* mutant (Fig. 11c). On the other hand, the double mutant ∆*dbpA*∆*srmB* displayed a cold-sensitive phenotype similar to that of the single mutants ∆*dbpA* and ∆*srmB*. This indicates that, although both genes are important for growth at low temperature, there is no additive effect on the severity of the cold-sensitive phenotype in the double mutant ∆*dbpA*∆*srmB* when both helicases are missing from the cells.

The effects of *rhlB* deletion in various helicase mutant backgrounds was analyzed by comparing the growth profiles of ∆*rhlB*, ∆*rhlB* ∆*rhlE*, ∆*rhlB*∆*srmB*, and ∆*rhlB*∆*rhl*E∆*srmB* strains at 22°C and 4°C (Fig. 11d and 12d). All these strains displayed normal growth at 22°C, and no appreciable differences were observed in their growth profiles (Fig. 12). At 4°C, ∆*rhlB* and ∆*rhlB*∆*rhlE* strains displayed wild type-like growth, suggesting that the two mutations exerted no additive effects, and both are dispensable for growth at low temperature singly or simultaneously. It is also important to note that the double mutant ∆*rhlB*∆*srmB* grew slowly at 4°C, similar to the cold-sensitive single ∆*srmB* mutant, suggesting that RhlB depletion did not exacerbate the growth defect of SrmB depleted cells (Fig. 11d). This was akin to the results obtained with RhlE depleted cells of ∆*srmB* in the ∆*rhlE*∆*srmB* double mutant (Fig. 11a). A summary of the interactions between the various helicase deletions of *P. syringae* is presented in Table 7.

**TABLE 7:** Genetic interaction among the helicase mutations of *P. syringae*, as judged from their combinational effects on the growth of the mutants at 4°C

	∆*csdA* (*csdA::spec* ^*R*^)	∆*srmB* (*srmB::kan* ^*R*^)	∆*dbpA* (*dbpA::spec* ^*R*^)	∆*rhlB* (*rhlB::spec* ^*R*^)	∆*rhlE* (*rhlE::tet* ^*R*^)
∆*csdA* (*spec* ^*R*^)	No growth at 4°C (*spec* ^*R*^)				
∆*srmB* (*kan* ^*R*^)	∆*csdA* ∆*srmB* (*spec* ^*R*^ *kan* ^*R*^) not obtained; probably lethal	Slow growth at 4°C(*kan* ^*R*^)			
∆*dbpA* (*spec* ^*R*^)	∆*csdA* ∆*dbpA* (Not tested)	∆*dbpA* ∆*srmB* (*spec* ^*R*^ *kan* ^*R*^); Slow growth like single-mutants: No additive effect	Slow growth at 4°C (*spec* ^*R*^)		
∆*rhlB* (*spec* ^*R*^)	∆*csdA* ∆*rhlB* (Not tested)	∆*rhlB* ∆*srmB* (*spec* ^*R*^ *kan* ^*R*^); Slow growth, like parental ∆*srmB*: No synthetic effect	∆*rhlB* ∆*dbpA* Not tested	WT-like; grow at 4°C (*spec* ^*R*^)	
∆*rhlE* (*tet* ^*R*^)	∆*csdA* ∆*rhlE* (*spec* ^*R*^ *tet* ^*R*^) Fully cold-sensitive like parental ∆*csdA*; No synthetic effect	∆*rhlE* ∆*srmB* (*tet* ^*R*^ *kan* ^*R*^); Slow growth like parental ∆*srmB*: No synthetic effect	∆*rhlE* ∆*dbpA* (*tet* ^*R*^ *spec* ^*R*^); Slow growth like parental ∆*dbpA*: No synthetic effect	∆*rhlE* ∆*rhlB* (*tet* ^*R*^ *spec* ^*R*^); WT-like: No synthetic effect	WT-like; grow at 4°C (*tet* ^*R*^)

Remarkably, severity of the cold-sensitive growth defect in the triple mutant ∆*rhlB*∆*rhlE*∆*srmB* (Fig. 11d) lacking RhlB, RhlE, and SrmB helicases simultaneously, was enhanced compared to the double mutants ∆*rhlB*∆*srmB* (Fig. 11d) and ∆*rhlE*∆*srmB* (Fig. 11a). The modest but marginal increase in the severity of the defect in the triple mutant at low temperature suggested that, although RhlB and RhlE helicases are ordinarily dispensable for the growth, they play certain subtle role in RNA metabolism, whose effects are manifested during the growth only when both are removed together with SrmB in the cells at low temperature.

### Functional complementation of RNA helicase mutant strains

Genetic complementation of the helicase deletion mutants was important for confirmation that the growth defects in the mutants are only due to gene disruption and not due to any other unexpected secondary alteration in the cells. This would also confirm the theoretical prediction based on genome analysis, that there will be no polar effects on downstream genes in the mutants due to disruption of the monocistronic helicase genes. Additionally, cross complementation of one helicase mutant with other helicases will provide an insight into the functional redundancy between the DEAD-box RNA helicases. Accordingly, each of the five helicase genes were cloned in the broad host range plasmid, pGL10, which can replicate in *P. syringae* Lz4W, and the helicase genes are constitutively expressed from the *lacZ* promoter of the pGL10.

**Fig 13:**
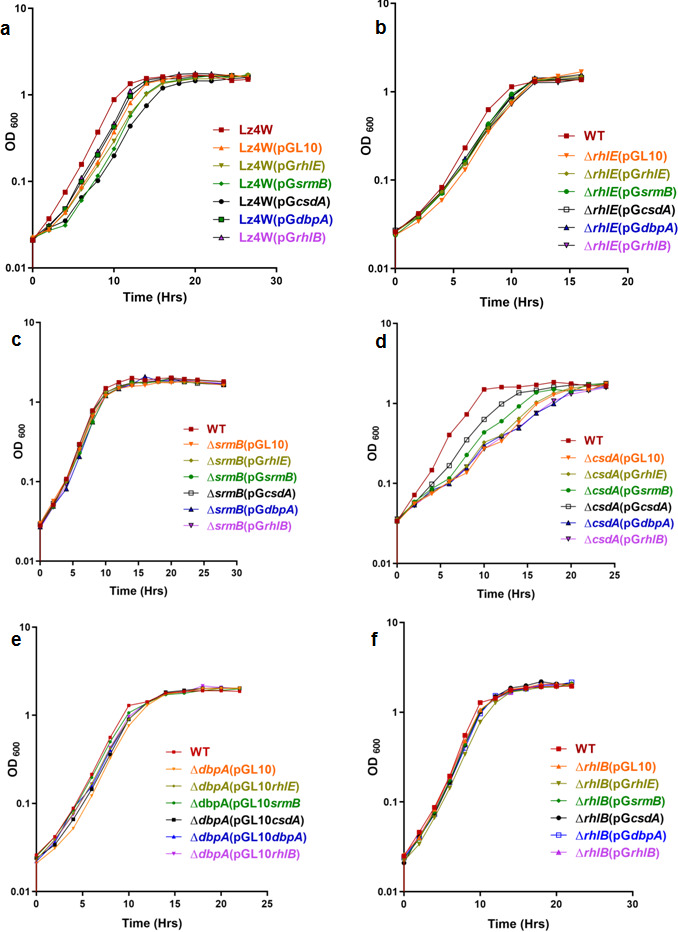
Functional complementation of helicase mutant strains: Growth analysis for over-expression of all five plasmid borne helicases in WT [Lz4W], ∆*rhlE,* ∆*srmB,* ∆*csdA,* ∆*dbpA and* ∆*rhlB* mutant strains at 22°C. Each growth curve has been performed at least three times All growth curves were generated using GraphPad Prism 4.0 software.

**Fig 14:**
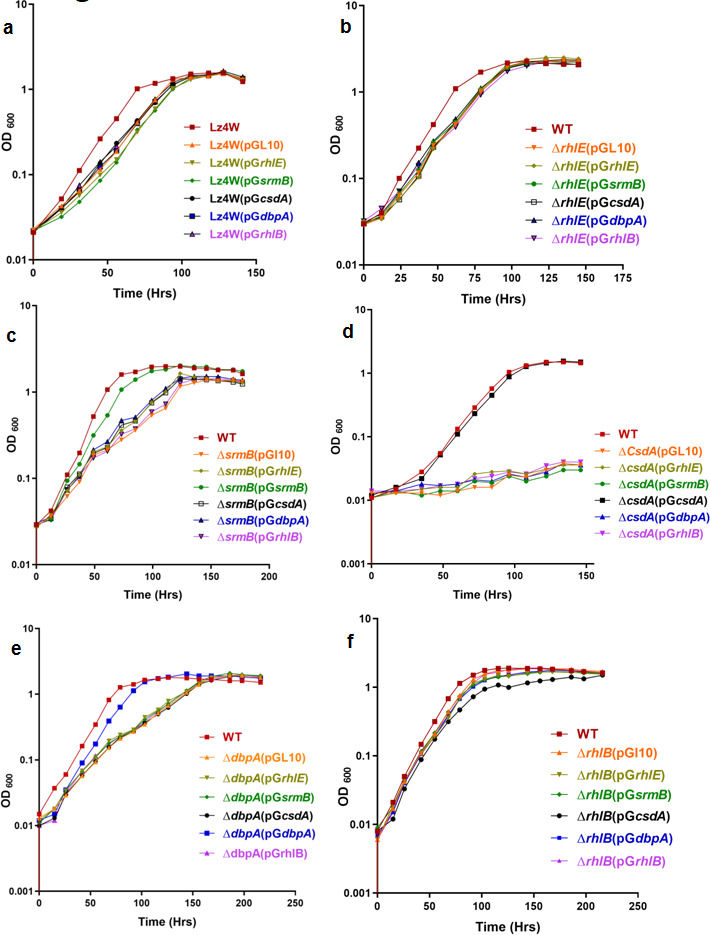
Functional complementation of helicase mutant strain. Growth analysis for over expression of all five plasmid borne helicases in WT [Lz4W], ∆*rhlE,* ∆*srmB,* ∆*csdA,* ∆*dbpA and* ∆*rhlB* mutant strains at 4°C. Each growth curve has been performed at least three times. All growth curves were generated using GraphPad Prism 4.0 software.

The complemented mutants were grown at 22°C and 4°C for analyzing their growth properties compared to the parental mutant strain. The 22°C and 4°C growth profiles of the complemented cold-sensitive strains ∆*csdA* (Fig. 13d and 14d), ∆*srmB* (Fig. 13c and 14c), and ∆*dbpA* (Fig.13e and 14e) show that the growth defects of the helicase mutants are rescued only by the cognate helicases expressed from the plasmids in the cell. The cold-sensitive phenotype of helicase mutants could not be rescued by expressing other helicases, suggesting that there is no cross complementation or any partial rescue of the phenotypes due to overlapping functions.

The complemented mutants ∆*rhlB* (Fig. 13f and 14f) and ∆*rhlE* (Fig.13b and 14b) harboring all six plasmids, including the empty pGL10, also did not show any alteration in their growth due to overexpression of the RNA helicases from the plasmids. ∆*rhlB* and ∆*rhlE* mutants by themselves also did not display any growth defects at either temperature (22°C and 4°C). Growth analysis of the wild-type strain harboring the additional RNA helicase genes on plasmids did not reveal any effect either on the growth or on the viability of the cells at 22°C or 4°C (Fig.13a and 14a)

## DISCUSSION

Microbes respond to fluctuations in temperature by changing their physiological response to ensure survival and growth. The physiological response to temperature downshift includes reduced transcription, translation, and degradation of RNA molecules. The cells adapt to these unfavorable environmental changes by expressing cold-induced proteins (41 – 43). Despite low reaction rates, psychrophiles grow at freezing temperatures with rates that can be compared to growth rates of mesophiles at higher temperature. One of the adaptive measures in psychrophiles is the presence of enzymes with low activation enthalpies as compared to the mesophilic enzymes (44). Structural rearrangement of RNA is accompanied by large activation enthalpies and becomes slow at low temperature. Since low temperature enhances the stability of nucleic acid secondary structures, one of the essential requirements for growth at low temperature would be to make them functional by structural rearrangement so that the physiological process goes on at acceptable rates (45). Rearrangement of RNA molecules is assisted by DEAD-box RNA helicases, a family of ATP-dependent RNA-binding proteins where ATPase activity is stimulated by interaction with RNA. The energy released from ATP hydrolysis is used in modulating and/or structural rearrangement of RNAs. This requirement for rapid RNA rearrangement at freezing temperatures prompted us to study the role of DEAD-box RNA helicases in cold adaptation.

Genomic analysis revealed that RNA helicase genes are not clustered in a specific chromosomal segment but dispersed all over the bacterial chromosome. The helicase genes are monocistronic and regulated by their independent promoters and transcription termination signals. The nucleotide sequence of the regulatory region of these genes did not throw up any noticeable sequence motifs that are specific for these genes or their expression at low temperature. Only the *csdA* transcript was observed to have a long 5′-UTR (227 bp) similar to the low temperature-specific transcript of *rhlE* that had 213-bp-long 5′-UTR. However, the two genes did not have any common regulatory sequences which can be correlated to their expression or their significance in the cells. Nonetheless, the monocistronic gene organization of helicase genes helped us to create gene knockout mutants by employing the antibiotic resistance cassette insertion without affecting the downstream gene expression (polar effects).

**Fig 15:**
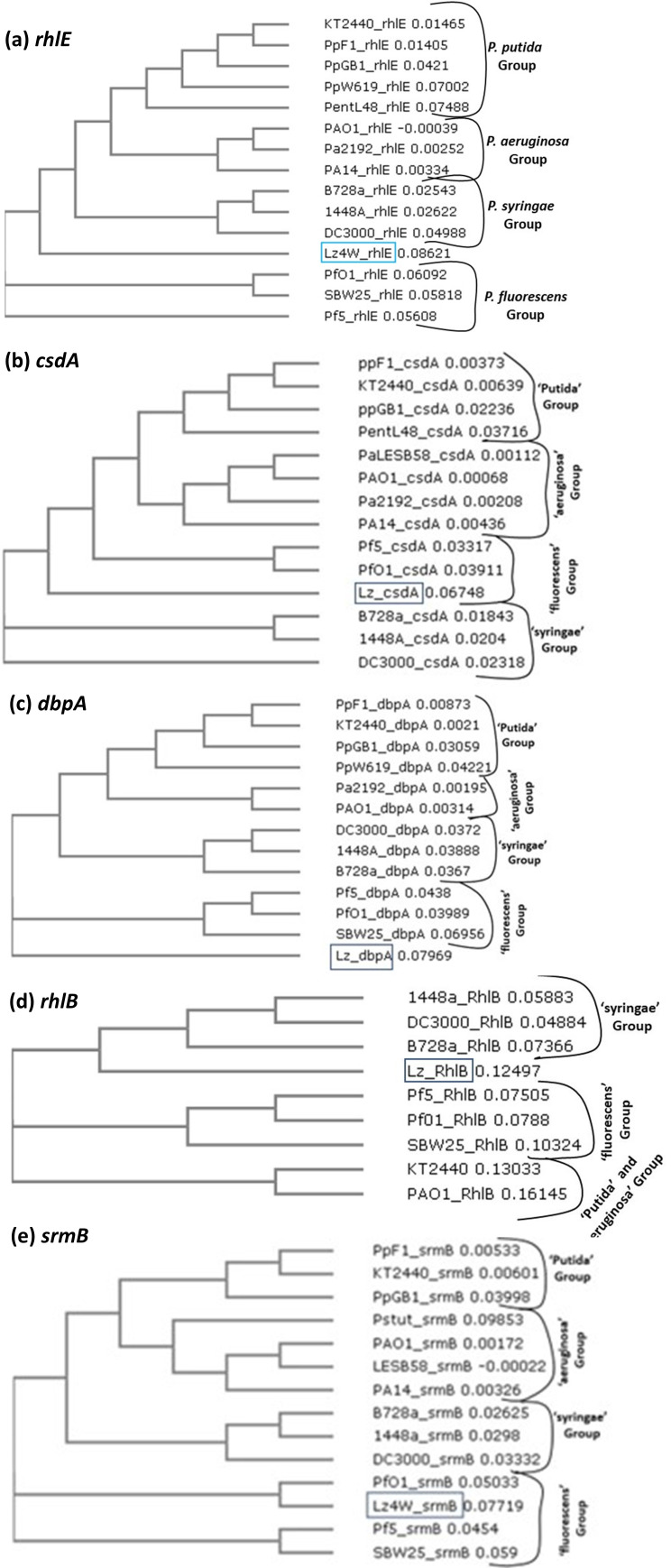
Gene sequence-based phylogenetic relationships among the DEAD-box helicase genes of the selected groups of *Pseudomonas* species. (a) *rhlE*, (b) *csdA*, (c) *dbpA*, (d) *rhlB*, and (e) *srmB* genes. The ClustalW program was used for multiple sequence alignment of the RNA helicase gene homologs from the representative strains of different *Pseudomonas* species. Neighbor-joining (NJ) trees were constructed from the default parameters at the www. ebi.ac.uk site. The program uses the NJ method for clustering the related species based on sequence identity and generates the phylogenetic tree. The numbers following the species indicate the evolutionary distance that has been calculated from the number of base substitutions per site. The strain names for different species are indicated on the trees. Accordingly, *P. putida* strains are indicated as pp KT2400, F1, GB1, and W619. The *Pseudomonas entomophila* L48 strain is shown abbreviated by pent L48. *P. aeruginosa* strains are indicated by PAO1, Pa2192, and PA14. The phytopathogenic *P. syringae* spp. are represented here by the strains B728a,1448a, and DC3000. *P. fluorescens* spp. are represented by PfO1 and SBW25 strains. The strain Pf5 is now classified as *Pseudomonas proteagens* Pf5 and not as *P. fluorescens* strain.

A gene sequence-based phylogeny of the *Pseudomonas* DEAD-box RNA helicases was performed. Homology-based sequence alignments of RNA helicase genes were performed to examine the species-specific divergence among genes and various *Pseudomonas* groups (Fig. 15). The helicase genes *rhlE*, *csdA*, *srmB*, *dbpA*, and *rhlB* of different species are broadly clustered in four distinct groups representing *fluorescens*, *putida*, *syringae*, and *aeruginosa* clusters. The most closely related group of *P. syringae* Lz4W is that of the *Pseudomonas fluorescens* group with ~84% to 85% identity in nucleotide sequence; the second group is represented by the *Pseudomonas syringae* group to which Lz4W shows ~81% identity; the third group is represented by the *Pseudomonas putida* group to which Lz4W shows ~75 to 76% identity; and the fourth group is represented by the *Pseudomonas aeruginosa* group to which Lz4W sequence shows only ~72% to 73% identity. Thus, RNA helicase branches of *Pseudomonas syringae* Lz4W and *Pseudomonas aeruginosa* have probably diverged much early in the pseudomonad evolution, while those for the Lz4W and *fluorescens* groups might have branched off relatively recently. The closer sequence identity to the Lz4W and *fluorescens* groups of pseudomonad species than to the plant pathogenic *syringae* group of species is noticeable. However, it is important to note that the laboratory analysis of the *P. syringae* Lz4W genome sequence has suggested that the Lz4W strain is a novel cold-adapted species of the *Pseudomonas* genus and is not a member of the *P. syringae* group (24, 46).

Analysis of the single-deletion mutants of helicase genes has shed new light on the role of the individual RNA helicases in the growth of *P. syringae*. Our study shows that three of the five DEAD-box RNA helicases are important for growth of the psychrophilic bacterium at low temperature (4°C). These three helicases are SrmB, CsdA, and DbpA, all of which are known to affect the biogenesis of the 50S ribosomal subunit and hence mature 70S ribosomes in *E. coli* (15, 16). Interestingly, the *srmB* and *csdA* deletions in mesophilic *E. coli* also led to a cold-sensitive phenotype in the bacterium, in which ∆*srmB* and ∆*csdA* mutants could grow at 25°C (low temperature for the mesophile) with a longer generation time (~90 and 138 minutes, respectively) compared to the wild type (generation time 77 minutes) (12). The ∆*dbpA* did not show any cold-sensitive growth defect at 25°C. These are in contrast to our observation with the psychrophilic *P. syringae*, in which *csdA* is absolutely essential, and the *srmB* or *dbpA* genes are important for the growth at low temperature (4°C). The ∆*csdA* mutant did not grow at all, while ∆*srmB* and ∆*dbpA* mutants grew slowly at the low temperature. In this respect, the requirement of *dbpA* and the absolute necessity of *csdA* for growth of the psychrophile at low temperature (4°C) are novel findings of this study.

At optimal temperature for growth, the requirement of DEAD-box RNA helicases has not been reflected by mutational studies in bacteria, especially using the single-deletion mutants. At 37°C, all single helicase-deletion mutants of *E. coli* exhibited ~25.5-minute generation time, similar to wild type (12). Interestingly, the ∆*csdA* mutant of *P. syringae* demonstrated the importance of *csdA* at the optimal temperature (22°C) of growth for the psychrophilic bacterium. ∆*csdA* showed a substantial increase in the generation time (3.97 hours) as compared to wild type (generation time 2.02 hours). All other four single-deletion mutants for DEAD-box RNA helicases (∆*rhlE*, ∆*srmB*, ∆*dbpA*, and ∆*rhlB*) did not show any growth defect or alteration in the viability at optimum temperature (22°C). These results suggested that the various RNA folding pathways and the RNA secondary structures are optimized for functions at the temperature in which bacteria grows at a maximum rate. The lack of any particular RNA helicase is probably not felt unless the RNA helicase is necessary for a very specific function that affects cell viability or cell growth. *csdA* probably serves a similar function in *P. syringae*, as a result of which the ∆*csdA* mutant grows slowly at 22°C and fails to grow due to cellular lethality at lower temperature (4°C) (47).

The loss of cell viability at low temperature due to RNA helicase deficiency was observed not only with ∆*csdA* but also in two other cold-sensitive mutants (∆*srmB* and ∆*dbpA*) of *P. syringae*. These mutants grew slowly (generation time 13.01 hours for ∆*srmB* and 12.6 hours for ∆*dbpA*) compared to the wild type (generation time 6.75 hours) at 4°C, suggesting that the slow-growing phenotype could be partly related to the cell death observed in the mutants at low temperature. The growth rates of both ∆*srmB* and ∆*dbpA* mutants are proportionate (generation time 13.01 hours for ∆*srmB* and 12.6 hours for ∆*dbpA*), but the degree of cell lethality associated with ∆*srmB* mutant is more as compared to cell lethality associated with the ∆*dbpA* strain (Table 6). The reduced cell death displayed by the ∆*dbpA* mutant may provide an important clue regarding the role of *dbpA* in cell growth. The absence of the *dbpA* gene has only a static effect on growth of the *dbpA* disrupted *P. syringae* mutant. The slower RNA metabolism in the absence of RNA helicases is likely to affect the growth rate, mostly at the low temperature. The role of *csdA* and *srmB* in the low-temperature growth of mesophilic *E. coli* has been reported earlier; however, the importance of *dbpA* in the cold-adapted growth of *P. syringae* is a unique finding of the study and needs to be investigated further.

Due to functional redundancy of gene products, especially in multigene families, the mutant phenotype of the cells is not manifested sometimes in the single-deletion mutants. The double-deletion mutants were constructed using the disrupted alleles of Δ*rhlE*, Δ*srmB*, Δ*csdA*, Δ*dbpA*, and Δ*rhlB*Δ*csdA*, which indicated that there is not much additive or synergistic effects due to their combination in the cells, except for the double mutants Δ*srmB*Δ*csdA* and Δ*dbpA*Δ*rhlE*. The Δ*srmB*Δ*csdA* mutant could not be recovered in our experiments, possibly due to the combinatorial lethality. On the other hand, the double mutant Δ*dbpA*Δ*rhlE* grew marginally better than the slow-growing Δ*dbpA* at the low temperature (4°C), suggesting a possible interaction between RhlE and DbpA on an unidentified common substrate (RNA) in the cells, in which RhlE exacerbates the effects of DbpA depletion (in Δ*dbpA*). When RhlE is removed from such cells, as in Δ*dbpA*Δ*rhlE*, the deleterious effect of DbpA depletion is marginally relieved in the double mutant. We also observed that, although Δ*rhlB*Δ*rhlE* does not have a growth defect, and Δ*rhlB*Δ*srmB* and Δ*rhlE*Δ*srmB* combinations do not show ameliorating (synthetic rescue) or worsening (synthetic lethality) of the cold-sensitive phenotype of Δ*srmB*, the triple-deletion Δ*rhlB*Δ*rhlE*Δ*srmB* exacerbates the cold-sensitive growth of Δ*srmB*. These results suggest that the cellular lack of RhlB or RhlE activities is not manifested in the cell growth, possibly due to their subtle roles in cellular milieus of Δ*srmB*, but when all of them are combined in the Δ*srmB* background, their requirements are manifested in the growth rate of the mutant, which slows down further at the low temperature. The results of these mutational studies thus point toward the existence of different types of subtle and not-so-subtle interactions among the RNA helicases in *P. syringae*.

Microscopic studies of the DEAD-box RNA helicase depleted cells suggest an important role for the RNA helicases in the regulation of cell size, morphology, and cell survival as evidenced by the alteration in cell morphologies and cell lethality of the cold-sensitive ∆*csdA*, ∆*srmB*, and ∆*dbpA* mutants at low temperature. However, helicase deficiency-associated cell lethality and changes in morphology may be directly through abrogation of ribosome function, defective RNA processing, or indirectly impacting the cellular pathways that regulate cell shape, size, and survival. In this context, exposure to low temperature causes a uniform decrease in size of all mutant strains except ∆*dbpA* mutant cells. ∆*dbpA* cells display only a marginal reduction in size as compared to other mutants (Table 8) (26, 48) Reduction in cell size on being exposed to challenging temperatures might be due to restructuring of the cell wall or realignment of membrane lipids and may be crucial for cold adaptation. Microscopic studies have revealed that the ∆*dbpA* mutant displays only a marginal size reduction at low temperatures, which may be attributed to the absence of the dbpA mutant. The role of dbpA in membrane restructuring and cell size regulation needs to be further studied.

**TABLE 8:** Cold induced decrease of cell size in *P. syringae^a^*

*P. syringae* strain	Size in microns	
	22°C	4°C
WT	2.10 ± 0.15	1.58 ± 0.20
∆*rhlE*	2.05 ± 0.2	1.55 ± 0.15
∆*srmB*	2.16 ± 0.15	1.55 ± 0.17
∆*csdA*	2.17 ± 0.14	1.61 ± 0.10
∆*dbpA*	2.02 ± 0.12	1.90 ± 0.13
∆*rhlB*	2.07 ± 0.2	1.57 ± 0.22

^*a*^ The data represent the measured cell sizes of *P. syringae* RNA helicase mutants at ambient [22°C] and low temperature [4°C]. The measurements were performed by imageJ software. The size of each cell mentioned here is the mean value of 7–10 cell size measurements.

One of the novel roles of DEAD-box RNA helicases we reported recently was that of providing protection to *P. syringae* against oxidative stress and UV- and mitomycin C-induced DNA damage, pointing toward the role of these enzymes in DNA repair and maintenance of genome integrity (49). CsdA was found to provide protection against UV radiation and paraquat, whereas SrmB provided protection against hydroxyurea, which causes damage to replication forks by dNTP depletion and induces oxidative stress in cells. The RNA helicases RhlE, DbpA, and RhlB do not seem to have any role in protection against any of the tested DNA-damaging agents (UV, mitomycin C, paraquat, H_2_O_2_, and hydroxyurea). On the other hand, SrmB and CsdA are important for low-temperature adapted growth and for protection against some of the DNA-damaging agents. Interestingly, ∆*srmB* and ∆*csdA* mutants display cold sensitivity to different extents and also display sensitivity toward different types of DNA-damaging agents. The requirement of RNA helicases for protection against these agents may lie either in prevention of cellular damage or repairing the damage once it occurs. DNA-damaging agents also cause damage to the RNAs, especially by oxidative base modification and RNA cleavage, leading to RNA damage-induced cellular lesions. Therefore, it remains to be elucidated how exactly RNA helicases play this protective role. The RNA helicases may be involved in transcription and translation of protective enzymes, removal of damaged RNAs, or in transcription coupled DNA repair, or directly by modulating RNAs that are involved in DNA repair by some unknown mechanism. To pinpoint the role played by individual RNA helicases, different substrates with which these helicases interact need to be identified.

Our results suggest that the DEAD-box RNA helicase *csdA* is absolutely necessary for the psychrophilic adaptation of the Antarctic *P. syringae*. The study also pointed out the importance of *dbpA* helicase in the cold adaptation of the psychrophilic bacterium, which binds to a specific region (helix 92) of 23S rRNA, but the deletion of which does not affect the growth of mesophilic *E. coli* at low temperature (17), The current study also pointed out the importance of *srmB* helicase at low temperature, which has been implicated in the assembly of 50S ribosomal particle in *E. coli* (15, 16). Thus, all the three DEAD-box RNA helicases which have been found to be important for growth of the Antarctic *P. syringae* at low temperature are known to participate in the 50S ribosomal subunit maturation and ribosome biogenesis in *E. coli* and *B. subtilis* (11, 14 – 17), suggesting the crucial dependence of ribosome assembly and protein synthesis on RNA helicases at low temperature. The important role of RNA helicase *srmB* and *csdA* in the cold-adapted growth of the Antarctic bacterium *Pseudomonas syringae* and the interesting role of these helicases in providing protection against DNA-damaging agents and oxidative stress need to be further investigated. Exploring the dependence of cellular process on RNA helicases through abrogation of ribosome function, defective RNA processing, or their independent involvement in different pathways related to nucleic acid metabolism will be insightful and fascinating.
